# Text messaging with or without financial incentives versus a waitlist control for weight loss in men: cost-effectiveness analysis of the Game of Stones randomised controlled trial

**DOI:** 10.1016/j.lanepe.2025.101328

**Published:** 2025-05-21

**Authors:** Abraham M. Getaneh, Marjon van der Pol, Dwayne Boyers, Alison Avenell, Seonaidh Cotton, Stephan U. Dombrowski, Cindy M. Gray, Frank Kee, Lisa Macaulay, Michelle McKinley, Catriona O’Dolan, James Swingler, Claire Torrens, Katrina Turner, Graeme MacLennan, Pat Hoddinott

**Affiliations:** a Health Economics Research Unit, University of Aberdeen, UK; b Aberdeen Centre for Evaluation, University of Aberdeen, UK; c Centre for Healthcare Randomised Trials, University of Aberdeen, UK; d Faculty of Kinesiology, University of New Brunswick, Canada; e School of Social and Political Sciences, University of Glasgow, UK; f Centre for Public Health, Queen’s University Belfast, UK; g Faculty of Health Sciences and Sport, Pathfoot Building, Stirling University, UK; h Queen’s University Belfast, UK; i Centre for Academic Primary Care, University of Bristol, UK

**Keywords:** Obesity, Cost-effectiveness, QALYs, Weight loss, Text messaging, Financial incentives

## Abstract

**Background:**

Cost-effective weight loss interventions are needed for people with obesity, particularly men, who are less likely to engage with weight loss programmes. This study aimed to investigate the cost-effectiveness of text messaging plus financial incentives and text messaging alone compared to a waitlist control to help men lose weight.

**Methods:**

585 men with obesity were recruited to Game of Stones (GoS): a 3-arm randomised controlled trial in 3 UK areas. Text messaging alone participants received daily automated behavioural texts for 12-months (3% weight loss). Text messaging with financial incentives participants also received loss-framed financial incentives linked to achieving weight loss targets at 12-months (5% significant weight loss). A control group received no intervention for 12 months (1.3% weight loss) followed by 3 months of texts. We conducted a 24-month within-trial cost-effectiveness analysis and lifetime decision model from a UK NHS perspective. The PRIMEtime model extrapolated the impact of GoS weight-loss data on lifetime obesity related disease incidence, costs, and QALYs. Weight regain assumptions were explored in scenario analyses.

**Findings:**

Text messaging with financial incentives costs £243 and text messaging alone costs £110 per participant to deliver. There were no significant differences between 24-month total costs or QALYs across groups. When modelled over lifetime, the mean discounted QALYs per person were 12.48, 12.49, and 12.46 for text messaging with financial incentives, text messaging alone, and waitlist control, respectively. The corresponding mean discounted total costs per person were £15,277, £15,117, and £15,100. The between group results for text messaging with financial incentives versus control were: QALY difference (95% CI): 0.02 (0.007, 0.029); cost difference: £176 (£43; £311); Incremental cost-effectiveness ratio (ICER): £9748 (£7,705, £11,791). For text messaging alone versus control: QALY difference: 0.03 (0.015, 0.037); cost difference: £16.5 (-£117; £152); ICER: £628 (£-5,914, £5384).

**Interpretation:**

Text messaging with financial incentives and text messaging alone are cost-effective compared to waitlist control. Both are relatively low-cost interventions that can be scaled to improve weight loss for men. The optimal strategy between them depends on weight regain assumptions after 12 months.

**Funding:**

10.13039/501100000272National Institute for Health and Care Research (Ref: NIHR 129703). Trial Registration isrctn.org Identifier: ISRCTN91974895.

**Research in context**

**Evidence before this study**

We performed a scoping review to identify existing economic evaluations of text message-based interventions for weight loss in adult men. We searched PubMed and Google Scholar for peer-reviewed papers published from inception to November 2024 using the search terms “obesity”, “overweight”, “weight loss”, “text messaging”, “financial incentives”, “cost-effectiveness analysis”, and “economic evaluation”. We found no previous long-term cost-effectiveness analysis of text messaging with or without financial incentives to support weight loss in men with obesity. One study reported the cost-effectiveness of goal-directed and outcome-based financial incentives for weight loss, but the study did not include any extrapolation of results beyond the trial period. Understanding the long-term impact of weight loss on obesity related disease, cost and quality of life is important to determine the most cost-effective weight loss strategies.

**Added value of this study**

This study provides a comprehensive assessment of the cost-effectiveness of text messages, with or without loss-framed incentives, for weight management in men with obesity. The study is conducted and integrated within the largest trial of these interventions to date and uses decision modelling to extrapolate trial results over a lifetime horizon. We found that text messages, with or without financial incentives are cost-effective, compared to a waitlist control, in helping men with obesity lose weight. The most cost-effective of the two intervention depends on assumptions about long-term weight regain.

**Implications of all the available evidence**

People with obesity, especially men, who are less likely to participate in weight loss programmes, require cost-effective weight loss interventions. Text messages, with or without loss-framed incentives are a cost-effective strategy to help men with obesity lose weight. The optimal intervention overall depends on assumptions about weight regain over time and further long-term data are required to validate these modelling assumptions. Future work should compare the cost-effectiveness of these interventions against other public health measures.

## Introduction

Obesity related diseases such as type 2 diabetes, heart disease, stroke, and some cancers lead to premature mortality and negatively impact quality of life.^1^ The global annual societal cost of obesity has been estimated at about $2.0 trillion.^2^ Obesity, defined as having a body mass index (BMI) ≥30 kg/m^2^, affects approximately 16% of people worldwide.^3^ In the UK, adult male obesity rates are about 26%.^4^ Evidence shows that the excess risk of premature death among those who have overweight or obesity is about three times as great in men as in women.^5^ Men, however, are less likely than women to take part in weight loss interventions.^6^^,^^7^ To date there have been few evaluations of the effectiveness and cost-effectiveness of interventions targeted towards men. Text message delivered behaviour change interventions and financial incentives have been proposed as ways to help men lose weight.^8^, ^9^, ^10^, ^11^, ^12^

The Game of Stones (GoS) trial was a multi-centre, parallel-group, 3-arm randomised controlled trial (RCT) that examined whether an intervention that combined text messaging with loss-framed financial incentives attained significant weight loss at the 12-month follow-up compared with the control group and whether an intervention of text messaging alone attained significant weight loss at the 12-month follow-up compared with the control group. The primary outcome results at 12 months showed a statistically significant weight loss between the text messaging with financial incentives group and the control group but not between the text messaging alone group and control.^13^

Despite evidence of weight loss benefit, especially for the text messaging with financial incentives group, it is crucial to also consider cost-effectiveness when informing widespread rollout of weight loss interventions. This paper provides a comprehensive assessment of both the short (within trial analysis) and long-term (economic model) cost-effectiveness of text messaging with financial incentives and text messaging alone to help men with obesity lose weight. Such assessments are rarely reported but are crucial to ensure that scarce healthcare funding is spent wisely, to maximise patient benefit.

## Methods

### The Game of Stones trial population

Full details of the GoS trial, including protocol amendments, are published elsewhere.^13^^,^^14^ The protocol was adhered to and all amendments were approved by the trial steering committee and the ethics committee. Briefly, N = 585 adult men with obesity, BMI ≥30 kg/m^2^, were recruited to the GoS study between July 2021 and May 2022 through community information, General Practices and social media. The sample size calculation is detailed in the main trial report.^13^ All participants were weighed by fieldworkers using standardised procedures, and men completed study questionnaires at baseline, 12 months, and 24 months post randomisation. Ethical approval was provided on 11/December/2020 by North of Scotland Research Ethics Committee 2 [20/NS/0141]. All participants provided written informed consent.

### Interventions

Participants in the text messaging alone arm received daily texts for 12 months with evidence and theory-based behaviour change techniques embedded. Participants in the text messaging and financial incentives arm received the same text messages with loss-framed financial incentives. The full incentive endowed (£400) at the start was paid at 12 months if all verified weight loss targets from baseline were met (5%, 10% and 10% at 3, 6, and 12 months respectively). Money was deducted for each unmet target. Participants in the intervention groups were weighed at baseline, 3, 6 and 12 months. All participants, regardless of trial arm, were given pedometers to encourage self-monitoring and had access to a GoS website with links to evidence-based weight loss information and support for men’s health (e.g., Men’s Health Forum).^15^ The wait list control group received 3 months of text messages after collection of the primary outcome at 12 months (months 13–15) (in response to ethics committee feedback).

### Economic evaluation

A within-trial intention to treat cost-effectiveness analysis is reported using 24-month trial data and a decision model extrapolated the impact of weight loss on obesity-related disease incidence, costs, QALYs and cost-effectiveness over a modelled lifetime horizon. All analyses were performed from a UK National Health Service (NHS) perspective. Costs and QALYs accruing beyond year 1 were discounted by 3.5% per annum in line with best practice guidance.^16^ Reporting of this study followed the Consolidated Health Economic Evaluation Reporting Standards (CHEERS) reporting guideline.^17^

### Within trial analysis

#### Resource use & costs

Intervention costs were derived from participant level resource use that included frequency of text messages, financial incentives received, the staff and equipment cost of weighing participants, fixed costs of hosting the text delivery software, and the linkage to the weight database necessary for the participant notification texts for targets met. Use of GP, nurse, accident and emergency, outpatient and inpatient services were collected using participant questionnaires. Resource use from the trial was multiplied by national average unit costs, provided in Supplementary Table S1, to calculate total costs, reported in 2021/22 UK pound sterling (£).

#### Outcomes

The within-trial analysis reported incremental cost per Quality-Adjusted Life Year (QALY) gained and per 1% of weight loss achieved. Participant self-reported EQ-5D-5L data were mapped to EQ-5D-3L following the Ben van Hout algorithm and valued using the UK population 3L tariffs.^18^ QALYs were estimated using area under the curve based on linear extrapolation between measurement time points.

### Statistical analysis

Costs, weight loss, and QALY data were reported descriptively, and Generalised Linear Models (GLM) were applied to estimate between-group differences, for the intention to treat analysis set. Models were adjusted for baseline measures, study centre, and participant recruitment method. A modified Parks test and link test were used to choose the appropriate family (distribution) and link function, respectively. Accordingly, a gamma distribution with identity link function was used for total cost; a Gaussian distribution with identity link function was used for weight loss; and a beta distribution was used for QALY.

Missing data were imputed using multiple imputation with chained equations, assuming that the data were missing at random (base case). The proportion of missing data were as follows: Utility index at baseline, 12 months, and 24 months (% missing = 1.4%, 28.5%, and 40.7%); total costs at baseline, 12 months, and 24 months (10.8%, 35.4%, and 43.8%); and weight measurements at 12 months and 24 months (27%, 36%). Baseline weight, study centre, recruitment method, height, age, Index of Multiple Deprivation (IMD), and comorbidity status were used as predictors in the imputation model. The impact of missing data assumptions was explored in scenario analyses. Statistical analyses were conducted using Stata, version 16.1.

Incremental cost-effectiveness ratios (ICER) were calculated as cost differences between arms divided by benefit differences (QALYs or % weight loss). For interventions that improve health outcomes, ICERs <£20,000 - £30,000 per QALY gained can be considered good value for money.^16^ Results were presented using both a pairwise comparison for each intervention against control, and a full incremental analysis was used to determine the optimal intervention overall. Any strategy either strongly or extendedly dominated was removed before calculating the ICER. A strongly dominated strategy is one where the intervention is more costly but less effective than the comparator, whereas an extendedly dominated strategy is a one with an expected ICER greater than that of the most effective alternative.^19^

Non-parametric bootstrapping with 1000 iterations was used to illustrate uncertainty on the cost-effectiveness plane and to calculate cost-effectiveness acceptability curves (CEACs). The 95% CI was obtained by summarising the bootstrap results as follows: first, the sample mean was computed for each bootstrapped sample and ranked from low to high. Using the means at the 2.5% and 97.5% percentiles 95% confidence interval was determined. The location of the 97.5th percentile is (0.975) × (N + 1), while the 2.5th percentile is at (0.025) × (N + 1). If any of the numbers are not integers, they were rounded to the nearest integer.^20^ CEACs illustrate the probability of the alternative interventions being considered cost-effective, based on net monetary benefit, using a range of different thresholds of willingness to pay (WTP) for a QALY gained. ICERs were also reported for cost per percentage weight loss achieved at 2 years.

### Lifetime analysis

The PRIMEtime Cost-Effectiveness (PRIMEtime-CE) obesity model was used to calculate the impact of short-term weight loss from the trial on lifetime horizon expected costs, QALYs and cost-effectiveness (incremental cost per QALY gained) of each intervention. It has been described in detail elsewhere.^21^ Briefly, PRIMEtime-CE-obesity is a proportional, multistate life table population model. It calculates the impact of weight loss on obesity related disease incidence (coronary heart disease, stroke, type-2 diabetes, and cancers of the colon, liver, kidney, and pancreas), life-years (LYs), quality-adjusted life-years (QALYs) and health care costs for the UK adult population up to age 100 years. The model has been developed using R software and is openly available at GitHub - seamuskent/PRIMEtime-CE-Obesity.

### Model inputs

The model was populated with obesity related disease risk data, health state costs and utilities derived from disease registries and published literature. Supplementary Tables S2 and S3 give an overview of the model input parameters and sources. The Intervention’s effect (absolute weight loss at 12 months) and delivery costs per person for each arm (text messaging alone, text messaging with financial incentives, and weight list control) were derived from the GoS trial.

### Treatment effect beyond the trial period (12 months)

Intervention weight loss was assumed to be regained linearly until baseline weight is reached, according to the rate of regain between 12 and 24 months, using multiple imputation of missing weight measurement data from the trial (Supplementary Table S2). Weight regain for the control group was obtained from a recent systematic review^22^ rather than the trial data. This is because the trial control group received text messages for three months after the end of the 12-month intervention period which were unlikely to be generalisable to UK practice. The most appropriate weight regain assumptions were uncertain and four alternative assumptions are explored in scenario analyses. First, it was assumed that baseline weight is regained over 5 years. This assumption is commonly applied in the literature.^22^^,^^23^ Secondly, it was assumed that a 10% weight loss at 12 months is maintained beyond 5 years.^21^ Thirdly, the baseline weight carried forward method was used for missing weight data from the trial, as multiple imputation may be biased if data are missing not at random. In the final scenario, the weight regain value was estimated using complete case data (weight measurements available at both 12 and 24 months). A threshold analysis was conducted by varying the baseline weight regain value of the text messaging with financial incentives to determine the weight regain value that would be required to change the cost-effectiveness conclusions regarding the optimal treatment overall.

### Sensitivity and scenario analyses

A probabilistic sensitivity analysis, using 500 simulations, was performed to explore the impact of uncertainty around health state costs, utilities, the link between BMI and disease incidence and mortality, and the effect of text messaging alone and text messaging with financial incentives on weight loss at 12 months on cost-effectiveness results (Supplementary Tables S2 and S3). The 95% uncertainty (credible) intervals were obtained as the values ± 1.96 × SE on either side of the mean from the Monte Carlo simulation output. One-way deterministic sensitivity analyses were undertaken to explore the most important model parameter for cost-effectiveness. This was done by varying intervention costs, disease costs, health utility values, and weight regain times using the upper and lower bounds of their corresponding 95% uncertainty intervals. We also tested the model by discounting health outcomes at a lower rate of 1.5% per year and applied different weight regain assumptions described above. Sub-group analysis by BMI and age was also conducted. Analyses were conducted using R, version 4.2.2.

The health economic analysis plan is provided in the supplement (Supplement health economics analysis plan).

### Patient and public involvement

Patient and public partners were involved in all stages of the clinical trial design and development as well as monitoring trial progress on steering committees. Results of the work were communicated with stakeholders using policy briefs and updated results on the trial website.

### Role of the funding source

The Funder did not have a role in the design (beyond their review of the application), and conduct of the study; collection, management, analysis, and interpretation of the data; preparation, review, or approval of the manuscript; and decision to submit the manuscript for publication. The views expressed in this publication are those of the authors and not necessarily those of the NIHR or the UK government.

## Results

### Within trial analysis

The trial randomised 585 men of whom 374 (64%) were followed up at 24 months. 43% (text messaging alone group), 51% (text messaging with financial incentives group); and 54% (control group) provided complete cost data and 48% (text messaging alone group), 61% (text messaging with financial incentives group); and 59% (control group) provided complete QALY data.

Using multiple imputation of missing data, mean absolute weight change in Kg from baseline to 12 months was −5.79 (SD 8.76), −3.55 (8.83), and −2.01 (9.17) for the text messaging with financial incentives, text messaging alone, and control groups respectively. Mean absolute weight change in Kg at 24 months was −4.58(SD 8.9), 3.17 (8.0) and −3.42 (8.4) for the text messaging with financial incentives, text messaging alone, and control groups respectively. Compared with the control group, the mean percent weight change at 12 months was significantly greater in the text messaging with financial incentive group but not in the text messaging alone. The percent weight change at 24 months was not significantly greater either in the text messaging with financial incentives or text messaging alone compared to the control group.

Intervention costs, described in detail in Table 1, were £243 and £110 per participant for text messaging with financial incentives and text messaging alone respectively. For the text messaging with financial incentives group, 90/196 (46%) participants received incentives. The average incentives received per participant randomised to this arm of the trial was £128. There were no significant differences between groups for total costs up to 24 months, whilst total QALYs were slightly higher in the control group compared to text messaging alone. Full details of the within-trial analysis results are provided in Supplementary Figs. 1a and 2b and Tables S4–S10.

**Table 1:** Cost of delivering the GOS interventions.

Fixed cost	Units	Unit cost (£)	Total cost (£)
Programming text messaging	2 days	277.5 per day	555
Monitoring and supporting text messaging system	12 days	277.5 per day	3330
Dedicated telephone number	12 months	180 per 12 months	180
Hosting/supporting/maintaining network^b^	3 years	1333 per year	3999
Entering/editing phone numbers & monitoring start of text messages	2.5 h per week for 10.5 months	37 per hr^a^	4218
Screening text replies, changing frequency of text messages/updating phone numbers	3 h per week for 24 months	37 per hr^a^	11,544
Scales	4	315	1262.4
Height measure	4	92	368
Total fixed cost			25,456.4
Total fixed cost per participant			65.27
**Variable cost (cost per person)**			
Text messages	Up to 365	0.049 per text + 20% VAT	Up to 21.5
Weighing participants			
Baseline	20 min	0.55 per min^c^	11
3 months	10 min	0.55 per min	5.5
6 months	10 min	0.55 per min	5.5
12 months	20 min	0.55 per min	11
Printing costs			
Appointment card	1	0.04	0.04
Information about starting/stopping texts	1	0.105	0.105
Additional variable cost for text messaging with financial incentives			
Incentives			Up to 400
Administration costs (arranging bank transfer)	15 min	0.35 per min	5.25
Mock cheque	1	0.05	0.05
Incentive explanation	1	0.21	0.21
Average variable cost			
Text messaging alone			45
Text messaging with financial incentives			178
**Average total intervention costs**			
Text messaging alone			110
Text messaging with financial incentives			243

a Based on NHS band 4 administrator.^2^

b Hosting the text delivery software and the linkage to the weight database necessary for the participant notification texts for targets met.

c Based on band 3 NHS nurse.^2^ All the remaining information in the table is based on the GOS trial study.

### Economic modelling results

**Fig. 1:**
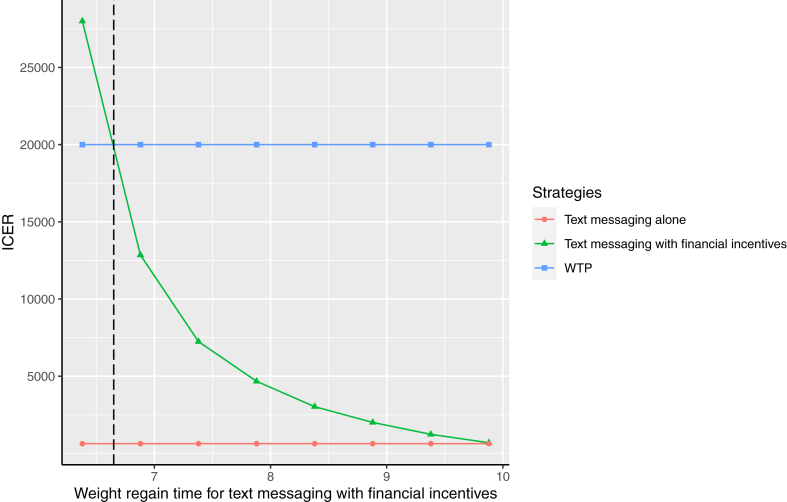
A threshold analysis diagram indicating the text messaging with financial incentives strategy becomes the optimal strategy with ICER below 20,000 when the weight regain time is slower from 4.9 years (base case) to 6.5 years.

Based on the weight loss trajectory observed in the trial, and assuming a linear rate of regain over time, we estimated that it would take 4.9 years for text messaging with financial incentives, 9.6 years for text messaging alone, and 7.2 years for control groups to return to baseline. The model predicted that, for every 100,000 people referred to text messaging with financial incentives, 205 (95% CI, 93; 314) incident coronary heart disease events, 43 (34; 120) incident strokes, 368 (306; 421) type 2 diabetes cases, and 11 (−54; 75) cancers would be avoided compared to control. For the text messaging alone, the corresponding predictions were 331 (217; 439), 66 (−12; 142), 625 (552; 674), and 24 (−41; 88) compared to control. In comparison to the control group, the economic model predicted that text messaging with financial incentives and text messaging alone both increased expected lifetime QALY gains, mean difference (95% CI) versus control: QALYs of 0.02 (0.007; 0.029) and 0.03 (0.015, 0.037) respectively. The additional discounted costs for text messaging with financial incentives and text messaging alone were £176 (£43; £311) and £16.5 (£-117; £152), respectively, compared to the control group. The corresponding incremental cost per QALY gained for text messaging with financial incentives and text messaging alone was £9748 (£7705; £11,791) and £628 (-£5914; £5384) respectively, the negative lower confidence bound for the text messaging alone represents cost saving. These make both strategies cost-effective compared to the control group. However, the full incremental analysis showed that text messaging alone was less costly and generated more QALYs than text messaging with financial incentives, suggesting that text messaging alone is the optimal strategy overall (Table 2, Supplementary Tables S11a and b). Subgroup analysis by BMI and age did not change the result (Supplementary Fig. S3). The cost-effectiveness acceptability curve from a probabilistic sensitivity analysis shows that the text messaging alone strategy has a high probability of being cost-effective (>90%) at a WTP threshold of £20, 000. The uncertainty around the ICERs is displayed in a cost-effectiveness plane (Supplementary Fig. S4a and 4b). Threshold analysis showed that when the weight regain time for text messaging with financial incentives was equal to or greater than 6.5 years, text messaging with financial incentives became the optimal strategy in the full incremental analysis, using a WTP threshold of £20,000, with an ICER of £18,562 per QALY gained (Fig. 1).

**Table 2:** Economic model cost-effectiveness results.

	Total Costs (£)	Total QALYs	Incremental costs versus Control	Incremental QALYs versus Control	ICER versus control (Pairwise)	ICER (Fully incremental)
**Base case**						
Control	15,101	12.47	–	–	–	–
Text messaging alone	15,117	12.49	17	0.026	628	628
Text messaging with financial incentives	15,277	12.48	176	0.018	9748	Dominated
**Scenario 1: equal weight regains time (5 years)**						
Control	15,126	12.46	–	–	–	–
Text messaging alone	15,198	12.47	72	0.010	7078	E. dominated
Text messaging with financial incentives	15,247	12.48	148	0.026	5721	4836
**Scenario 2: 10% of weight loss at 12 months maintained beyond 5 years**						
Control	15,126	12.46	–	–	–	–
Text messaging alone	15,144	12.49	18	0.032	555	555
Text messaging with financial incentives	15,185	12,52	59	0.061	973	1429
**Scenario 3: Missing weight measurement data imputed using the baseline weight carried forward method**						
Control	15,160	12.45	–	–	–	–
Text messaging alone	15,233	12.46	72	0.010	7130	7130
Text messaging with financial incentives	15,341	12.47	181	0.017	10,709	16,124
**Scenario 4: Observed data with only complete weight measurements at 12 and 24 months were used to calculate weight loss at the end of the interventions and weight regain at 2 years**						
Control	15,132	12.46	–	–		
Text messaging alone	15,161	12.48	29	0.022	1291	1291
Text messaging with financial incentives	15,261	12.49	130	0.030	4185	11,691

Both costs and QALYs are discounted at a rate of 3.5%. Total cost is the sum of intervention cost plus disease cost. Some numbers are rounded. E. dominated = Extendedly dominated.

### Scenario analysis

**Fig. 2:**
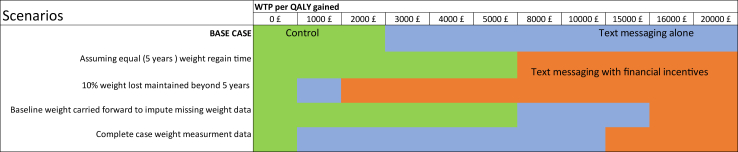
Preferred strategy in terms of cost-effectiveness for a range of willingness to pay (WTP) values. Preferred strategy is the strategy with the highest probability when compared to the other strategies considered. Strategies compared were (1) Wait list Control, (2) Text messaging alone, (3) Text messaging with financial incentives. A much faster weight regain time was used for text messaging with financial incentives in the base case analysis. In the base case, multiple imputation by chained equation was applied for handling missing weight measurements before calculating the weight regain.

The results are sensitive to the weight regain assumptions made in the model: (i) When using equal weight regain time for all three treatment arms (5 years), the text messaging with financial incentives strategy extendedly dominated the text messaging alone in the full incremental analysis with an ICER of £4836 per QALY gain (Table 2, Supplementary Tables S12a and b). (ii) Similarly, when it is assumed that a 10% weight loss at 12 months is maintained beyond 5 years, the optimal strategy in the full incremental analysis was text messaging with financial incentives with an ICER of £1429 per QALY gain (Table 2, Supplementary Tables S13a and b). (iii) When using the baseline weight carried forward method for missing data, text messaging with financial incentives was the optimal strategy with an ICER of £16,124 per QALY gain (Table 2, Supplementary Tables S14a and b). (iv) Finally, when using complete case data, text messaging with financial incentives was the optimal strategy with an ICER of £11,691 per QALY gain (Table 2, Supplementary Tables S15a and b). At a WTP threshold of £20,000, the text messaging with financial incentives strategy has a 70%–100% chance of being cost-effective, according to the probabilistic sensitivity analyses conducted in all the four scenarios (Supplementary Figs. S5a–8b). The scenario results are summarised in Table 2 and Fig. 2.

### One-way sensitivity analysis

The one-way sensitivity analysis results are displayed in tornado diagrams (Supplementary Fig. S9a and b). The weight regain times, intervention costs, and disease costs are the most influential variables. However, when these variables fluctuate within a specified range, the ICER is consistently below our pre-established WTP threshold, £20,000, suggesting that such fluctuations do not affect the model's output. The remaining variables have a relatively minor impact on the ICER.

## Discussion

In this study, we estimated the cost-effectiveness of text messages, with or without loss-framed incentives, for weight management in men compared to a waiting list control group. Despite evidence of weight loss in both the text messaging with financial incentives and text messaging alone groups over 12 months, these weight loss benefits did not translate to short term QALY gains. Whilst the non-significant QALY and costs differences between groups may be due to the study not being powered to detect cost-effectiveness differences between groups, it is more likely to be driven by the short time horizon being insufficient to capture all long term cost and QALY impacts of the impact of weight loss on a reduction in obesity related disease. Therefore, the results over the trial period should be interpreted cautiously as they do not reflect the long-term costs and health implications of the impact of weight loss on obesity related disease avoided. Obesity is related to various chronic diseases such as type 2 diabetes, heart disease, stroke, and some cancers, in which the benefits of weight loss in terms of avoiding these chronic diseases are generally realised further into the future, necessitating decision modelling to project the impact of weight loss over a lifetime horizon. Policy makers may therefore not be best informed by only considering cost per Kg lost from within trial cost-effectiveness analyses but cost per QALY gained from the lifetime decision model. Our within trial analysis reported an ICER (text messaging with financial incentives versus control) of £126 per 1% weight loss, which was comparable with Ladapo et al., 2024.^24^ To our knowledge this is the only within-trial economic evaluation comparable to our study.

The results of our economic modelling demonstrate the impact of reducing obesity-related disease in the longer term on cost-effectiveness. In contrast to the within trial cost-effectiveness analysis that did not show intervention QALY gains, the model predicted that both text messaging alone and text messaging with financial incentives lead to QALY benefits, and both are cost-effective compared to control over a lifetime horizon. The optimal strategy (text messaging alone or text messaging with financial incentives) was sensitive to the assumptions about weight regain over time. Our base case analysis, using trial measurement time points at 12 and 24 months, suggested a faster rate of weight regain for text messaging with financial incentives compared to text messaging alone. If that rate of regain was sustained longer term, text messaging alone would be the optimal treatment strategy. The faster weight regain rate in the text messaging with financial incentives group agrees with a systematic review and meta-analysis of behavioural weight loss programmes, reported by Hartmann-Boyce et al., in 2021.^23^ However, neither the GoS trial nor the individual studies included in the Hartmann-Boyce systematic review are long enough to fully capture the weight regain trajectory. Our cost-effectiveness threshold analyses show that text messaging with financial incentives would be the optimal strategy if it took at least 6.5 years to regain baseline weight. These findings suggest that maintenance of weight loss is key to achieving a cost-effective use of scarce NHS funding resources. Further research is required beyond the two years follow up of the trial to determine if it is feasible to maintain weight loss longer term with these interventions. Considering that 39% of the GoS trial participants came from low socioeconomic backgrounds and that residents of low-income neighbourhoods might be more receptive to incentives,^25^ one approach to maintain longer-term weight loss might be to offer modest booster incentives over an extended period. Offering appropriately timed or tailored text messages over the long term might also help to maintain weight loss.^26^ However, it is necessary to investigate the effect on the cost-effectiveness.

The strength of this economic evaluation is that it is informed by a multi-centre trial which enhances the generalisability of our results. This is the first study to estimate the cost-effectiveness of text messages with or without financial incentives over a longer period than typical for such interventions, for men with obesity. Ladapo et al. reported in 2024^24^ the cost-effectiveness of goal-directed and outcome-based financial incentives for weight loss in low-income populations. However, cost-effectiveness was estimated over the trial period only (12 months), and only incremental costs per kilogram lost were reported which is less useful to decision-makers because there is no explicit WTP threshold for weight loss. Another strength of the current study is, although we have not carried out a specific distributional cost-effectiveness analysis, the GoS interventions targeted men from poor socio-economic backgrounds where economic disparity associated with obesity is significant, and anti-obesity medications (AOMs) might incur higher out-of-pocket expenses.

Our study has some limitations. First, obesity has been linked to higher rates and costs of other medical conditions that are not covered by the PRIMEtime model, such as osteoarthritis in the knee and dementia.^27^^,^^28^ The healthcare savings that result from weight loss through the GoS interventions may therefore have been underestimated. Second, the model uses observational studies to estimate the effect of weight change on disease incidence and mortality. Estimates from observational studies are potentially biased, though there is strong Mendelian Randomisation evidence of such links being causal.^29^ Third, the weight regain time in the control group is based on data from a systematic review that includes studies from different parts of the world (mainly North America and Europe) and from both genders. This might introduce bias into the control group's real weight-regain time in the current study, which is specific to men population in the UK. The median age and BMI are, however, comparable to those in the GoS trial. In addition, the weight regain time for both the intervention arms is estimated based on only one year of follow-up data following the end of the interventions, and the model assumes that weight regains linearly for the rest of the years until weight returns to baseline; the same assumption applies for the control group. This may not reflect actual weight regain trajectories. In our scenario analysis, we tried to account for the impact of uncertainty surrounding the weight regain time. Future research would benefit from longer-term follow-up data on weight regain trajectories. This limitation, however, is not specific to our study. According to existing literature, one of the primary uncertainties regarding the long-term cost-effectiveness of other obesity interventions, such as AOMs is the absence of long-term weight-rebound rates following treatment discontinuation.^30^ Moreover, the long-term effect of adverse events associated with most AOMs' or surgical interventions on cost-effectiveness is often not assessed.^31^ Whilst a key strength of our work, decision modelling, by its nature of extrapolation over a lifetime horizon, requires simplifying assumptions to capture the costs and benefits of weight loss on obesity related disease in the longer term beyond trial follow up. For example, it is unclear whether losing weight and achieving a normal BMI equates the risk of long-term obesity related disease events to what would have been achieved if the weight was never gained in the first place. Another example is the long-term impact of weight regain on obesity related disease event risks. To quantify this uncertainty, we have conducted a wide range of scenario analyses to explore the impact of alternative, but plausible, assumptions on cost-effectiveness findings. We would therefore encourage readers to draw conclusions based on the totality of the scenarios provided.

Offering text messages with or without loss-framed incentives is cost-effective when compared to a waitlist control for men with obesity over the long term. The choice of the optimal strategy when a full incremental analysis is applied depends on assumed rate of weight regain. In the base case assuming faster weight regain for text messaging with financial incentives the text messaging alone was the optimal strategy, whereas in all the scenario analyses text messages with financial incentives were the optimal strategy.

## Contributors

Concept and design: van der Pol, Getaneh, Boyers, Hoddinott, O'Dolan, Macaulay, Dombrowski, Swingler, Avenell, Gray, Kee, McKinley, Torrens, Turner, MacLennan. Acquisition, analysis, or interpretation of data: Getaneh, van der Pol, Boyers, Hoddinott, O'Dolan, Macaulay, Dombrowski, Swingler, Cotton, Avenell, Kee, McKinley, Torrens, Turner, MacLennan. Drafting of the manuscript: Getaneh, Boyers, van der Pol. Critical review of the manuscript for important intellectual content: All authors. Obtained funding: Hoddinott, Dombrowski, Avenell, Gray, Kee, McKinley, Turner, MacLennan, van der Pol. Administrative, technical, or material support: Hoddinott, O'Dolan, Macaulay, Cotton. Supervision: van der Pol, Boyers. Getaneh and Boyers had access to the raw cost-effectiveness data for analysis. Getaneh performed the cost-effectiveness analyses and Boyers verified the accuracy of the analyses. All authors approved the decision to submit the manuscript for publication.

## Data sharing statement

The data collected for the study, including individual patient data for the within trial analysis and a data dictionary defining each field in the data set will be made available to others. The participant data will be de-identified and will comply with the ethical and regulatory approvals for the study. Requests for access to data can be sent by email to chart@abdn.ac.uk and will be considered by the study team.

## Declaration of interests

PH reported receiving grants from National Institute for Health Research (NIHR), and the Chief Scientist Office (CSO), Scotland, during the conduct of the study and serving as chair or member of Independent Trial Steering Committees unrelated to weight management trials; being a member of the NIHR School for Primary Care Research Funding panel. SD reported receiving grants from the NIHR and CSO during the conduct of the study. SC reported receiving grants from NIHR Public Health during the conduct of the study. AA reported receiving grants from NIHR Public Health during the conduct of the study. DB reported receiving grants from NIHR during the conduct of the study. MM reported receiving grants from NIHR and HSC R&D Northern Ireland during the conduct of the study and served as Advisory Council member (unpaid) for The Nutrition Society UK. CT reported receiving NIHR funding during the conduct of the study and serving as member of Executive Committee for UKSBM (unpaid). MP reported receiving grants from NIHR Public Health Research and the Chief Scientist Office, Scotland, during the conduct of the study. No other disclosures were reported.

## Appendix ASupplementary data

Supplement_health economics analysis plan
