# Supplementary material for: Text messaging with or without financial incentives versus a waitlist control for weight loss in men: cost-effectiveness analysis of the Game of Stones randomised controlled trial
Source: Lancet Reg Health Eur. 2025 May 21;54:101328. doi: 10.1016/j.lanepe.2025.101328 (PMC12149650; doi:10.1016/j.lanepe.2025.101328)
Supplement: Supplement_health economics analysis plan [file mmc1.docx]

**Health Economics Analysis Plan (HEAP) for the Games of Stones trial**

| **SAP version** | **Section number changed** | **Description of and reason for change** | **Health Economist** | **Date changed** |
| --- | --- | --- | --- | --- |
| **2.0** | **5** | **Minor changes to analysis of costs and outcomes to ensure consistency with statistical analysis.**  **Updated question used to measure use of NHS/local authority funded weight loss services and included a table for reporting these data.**  **Updated secondary objectives.** | **MvdP, AG** | **21.09/2023** |
| **3.0** |  | **Updated the analysis plan to include the 24 month data** | **MvdP, AG** | **27/06/24** |

**Section 1: Administrative information**

**Title:** Health economics analysis plan for the Games of Stones trial: a multi-centre randomised controlled trial to assess whether text and endowment incentives are effective and cos-effective for weight management in men with obesity

**Trial registration number:**

**Source of funding:** National Institute for Health Research, Public Health Research; Reference number – NIHR129703

**Purpose of HEAP:** The purpose of this HEAP is to describe the analysis and reporting procedure intended for the economic analyses to be undertaken. The analysis plan is designed to ensure that there is no conflict with the protocol and associated statistical analysis plan and it should be read in conjunction with them.

**Trial protocol version:** This document has been written based on information contained in the trial protocol version 2.0, dated 2 February 2021

**Trial Statistical Analysis Plan (SAP) version:** SAP Version: x.0, Date: 24 January 2021

**Trial HEAP version:** Version: 1.0, Date: 9 August 2021

**Roles and responsibilities:** This HEAP was prepared by Prof Marjon van der Pol. The trial health economists (Marjon van der Pol and a HERU Research Fellow (TBC)) are responsible for conducting and reporting the economic evaluation in accordance with the HEAP.

**Signature of person writing HEAP:
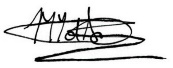
**

**Signature of Chief Investigator:**

**Section 2. Trial introduction and background**

**Trial background and rationale**

Obesity increases the risk of type 2 diabetes, heart disease, stroke, mobility problems and some cancers, and its prevalence in rising. Men engage less than women in existing weight loss interventions. This trial builds on our Game of Stones NIHR PHR-funded feasibility study.

**Aim of the trial**

1.Conduct a 3-arm RCT to estimate between group weight-loss difference at 12M for men with obesity who receive i) SMS+I; ii) SMS only; iii) 12M wait list for SMS 2.Assess differences between groups in secondary outcomes 3.Assess the cost-effectiveness of SMS+I and SMS only compared to a wait list control 4.Understand men's and service providers' experiences of the intervention 5.Follow up men at 24M (12M after intervention ceases) and request consent for linkage to long-term health outcome data 6.Refine the digital programming for future scalability and implementation.

**Trial population**

*Inclusion criteria*

- Men with BMI equal to or greater than 30kg/m^2^
- Aged 18 or over, understand study information and able to give informed consent.
- Resident in and around Glasgow, Belfast and Bristol.

*Exclusion criteria*

- Inability to understand the trial or the English language SMS intervention
- No mobile phone access
- Planning to move out of the area within 12 months
- Current or recent (in last 6 months) participation in a research weight loss intervention study (participants from the feasibility study are welcome to participate in this RCT)
- Plan to have bariatric surgery within 12 months.
- For GP screening prior to sending invitation letters
  - known terminal illness or severe psychiatric illness
  - known impaired cognitive or visual function that would limit understanding of study information and SMS.

**Objectives**

Are automated ‘Short Message System’ (SMS) texts, delivered to support behaviour change, with or without endowment Incentives (I), effective and cost-effective for weight change at 12M compared to a waiting list control in men with obesity?

Primary objective: To conduct a 3-arm RCT to estimate between group % difference in weight-loss at 12 Months (M) from baseline for men with obesity who receive i) SMS+I; ii) SMS only; iii) 12M wait list for SMS.

Secondary objectives:

- To assess differences between groups in secondary outcomes
- To assess the cost-effectiveness of SMS+I and SMS only compared to a wait list control
- To understand men’s and service providers’ experiences of the intervention
- To follow up men at 24M (12M after texts/incentives cease for the intervention groups; 9 months after the SMS cease for the waiting list group). Request consent for linkage to long-term health outcome data
- To refine the digital programming for future scalability and implementation.
- To compare PHQ-4, Warwick and Edinburgh Mental Wellbeing Scale (WEMWBS), Quality of Life (EQ-5D-5L-AD) Anxiety and Depression (AD) dimension and Weight Self-Stigma Questionnaire (WSSQ) measures for the 3 trial groups at baseline and 12 months
- To undertake exploratory moderator analyses examining interactions between mental health/wellbeing status at baseline and 12-month weight change
- To undertake exploratory mixed methods analyses examining mental health/wellbeing status, weight change trajectories, health inequalities, lived experiences and views to inform implementation, tailoring and future research
- To undertake exploratory moderator analysis for weight change at 12 months by the presence/absence of multiple long-term conditions, presence/absence of an obesity related comorbidity and presence/absence of diabetes at baseline
- To compare secondary Quality of Life, mental health/wellbeing outcomes for men with or without MLTC at baseline
- To undertake exploratory mixed method analyses to understand the lived experiences of men with MLTC and what would make a difference for recruitment, implementation, tailoring and future weight management interventions. Game of Stones Protocol v6.0 21.06.23 28
- To undertake a mixed method process evaluation to inform future implementation and research

**Interventions and comparators**

1) SMS ONLY: daily texts for 12M with evidence and theory-based behaviour change techniques embedded; 2) SMS + I: as above + financial incentives based on loss aversion theory. The full incentive endowed at the start is paid at 12M if all verified weight loss targets from baseline are met: 5% of weight lost at 3M, 10% lost at 6M and 10% lost at 12M. Some money is lost for each target that is not met. CONTROL: 12M wait list then SMS for 3M.

**Trial design**

Multicentre randomised controlled trial with three arms: text messages with incentive (SMS+I) text messages only (SMS); and waiting list for text messages (Control)

**Section 3. Economic approach**

**Aim of economic evaluation**

The aim of the economic evaluation is to address the question: are text message and endowment incentives cost-effective for weight management in men with obesity.

**Objectives of the economic evaluation**

To assess the cost-effectiveness of SMS+I and SMS only compared to a wait list control over trial period and modelled lifetime.

**Overview of economic analysis**

The within-trial economic evaluation will be performed using individual participant level data from the Games of Stones trial. The trial results will be extrapolated over a life-time horizon using the PRIMEtime-CE-obesity model [Kent S et al. 2019]. The analytical approaches will take the form of cost-utility and cost-effectiveness analysis. Incremental cost-utility (and cost-effectiveness) ratios will be calculated by taking a ratio of the differences in the mean costs and mean effects.

**Jurisdiction**

The trial is conducted in the UK which has a national health service (NHS), providing publicly funded healthcare, primarily free of charge at the point of use.

**Perspective**

The economic analysis will be from the NHS and Personal Social Service perspective.

**Time horizon**

The trial based economic analysis will compare the costs and consequences of each arm over the first 24 months after randomisation. The decision model will compare the cost and consequences of each arm over an extrapolated lifetime horizon.

**Section 4: Economic data collection & management**

**Statistical software**

Stata will be used for the within trial analysis. R will be used for the decision modelling.

**Identification of resources**

The cost categories included are: costs of the interventions (SMS and financial incentives); cost of primary and secondary care (GP, nurse, A&E, outpatient appointments and inpatient stays). Cost of NHS and local authority funded weight loss services will also be explored. The cost of the intervention includes: costs associated with sending SMS (equipment, consumables and staff time); and costs associated with the financial incentives (equipment, consumables and staff time).

**Measurement of resource use data**

NHS resource use (GP, nurse, A&E, outpatient appointments and inpatient stays) is measured as part of the participant questionnaire at baseline, 12 months and 24 months. Each question asks whether the participant has used a particular service and if so how many times. The questions ask about health service use in the last 3 months at baseline and in the last 12 months at 12 and 24 months. Use of NHS (or local authority) funded weight loss services clinics is also measured at 12 and 24 months. However, less detail is collected on this and the analysis of these data are more exploratory.

**Valuation of resource use**

Unit costs for NHS resource use are taken from standard sources. These are the PSSRU unit costs for GP and nurse contacts and Department of Health Reference costs for outpatient visits, A&E visits and inpatient stays. Unit costs of weight loss services subsidised by the NHS (or local authority) will be taken from the literature including Jolly et al 2011. The intervention costs are based on study specific estimates. Same base-year will be used for all costs in order to adjust for the effects of inflation. The most recent financial year for which all unit costs are available will be selected.

**Identification of outcomes**

The primary economic outcome will be Quality Adjusted Life Year (QALY) derived from utility scores measured using the EQ-5D-5L instrument. A secondary outcome measure is % weight loss.

**Measurement of outcome**

The EQ-5D scores are measured as part of the participant questionnaire at baseline and 12 months.

**Valuation of outcomes**

The EQ-5D data are translated into ‘utility scores’ using the UK population tariff. The current recommendation by the National Institute for Health and Care Excellence (NICE) is to map the EQ-5D-5L back to EQ-5D-3L and use the UK population tariff for the EQ-5D-3L. However, a new valuation study is currently being conducted and the new tariff will be used if available and recommended by NICE. QALYs are estimated by estimating the area under the lines that link the utility scores obtained at baseline, 12 months and 24 months.

**Section 5: Economic data analysis**

**Analysis population**

The full analysis set will include all randomised participants, which is in accordance with the “intention to treat” (ITT) principle.

**Timing of analyses**

The within trial analysis and the decision modelling will be conducted once all participants have been followed for 24 months after randomisation.

**Discount rates for costs and benefits**

Costs and benefits in the lifetime analysis will be discounted at 3.5% p.a. as recommended by NICE.

**Cost-effectiveness threshold**

The estimated mean QALYs and costs associated with each treatment option will be combined with a feasible range of values for decision makers’ willingness-to-pay (ʎ), to obtain the distribution of net benefits at different levels of ʎ. The primary economic analysis will use a cost-effectiveness threshold of £20,000 per QALY.

**Statistical decision rule**

Mean differences in costs, QALYs and net benefits between randomised groups will be estimated with associated 95% confidence intervals.

**Analysis of resource use**

Differences in the use of resources between randomised groups will be described but not compared statistically.

**Analysis of costs**

Incremental costs will be estimated using Generalised Linear Models (GLM) with appropriate distributions for cost data and adjustment for baseline cost, recruitment centre and recruitment route (GP and community)t.

**Analysis of outcomes**

Incremental QALYs will be estimated using Generalised Linear Models with adjustment for baseline EQ-5D, recruitment centre and recruitment route (GP and community).

**Missing data**

Trial data will be examined for any missing data. Completion rates will assess the number of participants for whom all items are missing and number of participants for whom some items are missing. The frequency and percentage will be reported for the NHS resource use and EQ-5D questions at all available time points and across the trial period. The appropriate method for dealing with missing data will depend on the proportion of missing data and likely mechanism of missingness. For example, multiple imputation methods may be used if the data is missing at random (MAR).

**Analysis of cost-effectiveness**

Cost and QALY data will be combined to calculate an incremental cost-effectiveness ratio (ICER) and net monetary benefit (NMB) statistic. Seemingly Unrelated Regression (SUR) will be used, if appropriate, to account for the correlation between the costs and the QALYs. Cost and %weight loss will also be combined to calculate an incremental cost-effectiveness ratio (cost per % weight loss).

**Sampling uncertainty**

Bootstrapping is used to estimate a 95% confidence interval surrounding the cost and QALY differences and the ICER. Results will be plotted on cost-effectiveness planes to illustrate the impact of sampling uncertainty on results.

**Subgroup analysis**

Analyses will also be conducted on the final dataset to investigate how cost-effectiveness varies between different subgroups in terms of deprivation (IMD 1,2 versus IMD 3,4,5).

**Sensitivity analysis**

Several scenario analyses will be undertaken to explore uncertainties surrounding key parameters in the economic evaluation. Cost-effectiveness will be estimated (1) using complete cases only; (2) varying the intervention costs; and (3) varying the unit costs of other resource use and (4) using last entry carried forward for missing values.

**Section 6: Modelling**

**Decision analytic modelling**

The trial results will be extrapolated over a life-time horizon using the PRIMEtime-CE-obesity model [Kent S et al. 2019].

**Model type**

The PRIMEtime-CE-obesity model is a proportional, multistate life table population model which links BMI to mortality and noncommunicable disease morbidity (type 2 diabetes, coronary heart disease, stroke, and cancers of the breast, colon, liver, kidney, and pancreas). It estimates disease incidence (coronary heart disease, any stroke, type-2 diabetes, and cancers of the breast, colon, liver, kidney, and pancreas), life-years (LYs), quality-adjusted life-years (QALYs), and health care costs for the UK adult population up to age 100 years.

**Treatment effect beyond the end of the trial**

The base case scenario is based on the weight regain observed between 12 months (end of the intervention) and 24 months. It is assumed that the same amount is regained in each of the subsequent years until weight is returned to baseline. Further scenarios will be modelled. Firstly, it is assumed that weight loss at 12 months is regained in a linear fashion between 12 months and 5 years following the end of the intervention). Secondly, it is assumed that a proportion of any weight loss (up to 1 kg) at 12 months is maintained beyond 5 years. Thirdly, weight regain assumptions are based on a recent systematic review by

by Hartman-Boyce (Hartmann-Boyce et al, 2021; Hartmann-Boyce et al, 2022) which showed that the weight regain after programme end was 0.12-0.32 kg/year greater in the intervention relative to control groups. The weight regain was faster (1 to 1.5 kg/year) for financial incentives. The midpoint will be used for the scenario.

**Methods for identifying and estimating parameters**

This is an existing model which has all relevant parameters identified and estimated. Treatment effects (% weight loss at 12 months) are derived from the trial data. The weight loss estimate will be updated if the study progresses to phase 3.

The model will be estimated for a hypothetical population with the same age, sex and deprivation characteristics as in the trial.

**Model uncertainty**

Parameter uncertainty will be assessed using probabilistic sensitivity analysis (PSA). In PSA all model steps are repeated 500 times using Monte Carlo Simulation. A distribution is defined for each parameter and a value is randomly drawn from that distribution for each of the 500 simulations. Outcomes are re-estimates using the randomly drawn parameters. The standard deviation of the estimated outcomes across bootstrap replicates are estimated and used to estimate a 95% confidence intervals assuming mean outcomes follow normal distributions

**Scenario analyses**

Several scenario analyses are undertaken to explore the impact of key assumptions on cost-effectiveness. These include: (1) varying the intervention costs; (2) discounting health outcomes at a lower rate (1.5%); l. Differences in Costs and QALYs will also be estimated for different time horizons (this will include estimates of any cost savings in NHS health care utilisation in the short, medium and long term) although we note that lifetime horizon is the most appropriate for decision making

**Section 7: Reporting/publishing**

CHEERS guidelines will be followed when reporting the health economic evaluation, in a format appropriate to stakeholders and policy makers.

**Deviations from the HEAP**

Any deviation from HEAP will be described and justified in the final published report.

**References**

Jolly, K. et al. Comparison of range of commercial or primary care led weight reduction programmes with minimal intervention control for weight loss in obesity: Lighten Up randomised controlled trial *BMJ* 2011;343: d6500

Kent, S., et al., Is Doctor Referral to a Low-Energy Total Diet Replacement Program Cost-Effective for the Routine Treatment of Obesity? Obesity (Silver Spring), 2019. **27**(3): p. 391-398.

**Section 8: Appendices**

**Health economics collection tools**

**Baseline**

| **Please look at the list of NHS Services below**  Please tick NO or YES. If you tick ‘yes’ for any of the services (these may have been provided face-to-face, by video or telephone), please give the number of times you have used the service in the LAST 3 MONTHS. This can be your best guess as the exact number may be difficult to remember.  *The example shows: two visits to the Dentist in last 3 months* | | | |
| --- | --- | --- | --- |
| **Over the LAST 12 MONTHS, have you used any of the following NHS Services?** | **No** | **Yes** | **Number of appointments (face-to-face, video or telephone)** |
| *Example: Dentist* | □ | 🗹 | **2** |
| GP/ Family Doctor | □ | □ |  |
| Nurse | □ | □ |  |
| Outpatient appointments | □ | □ |  |
| Emergency Care (casualty/A&E) | □ | □ | **Number of visits** |
|  |  |  |  |
| Inpatient stay | **No** | **Yes** | **Number of days spent in hospital** |
|  | □ | □ |  |

**12 month and 24 month questionnaire**

| **Please look at the list of NHS Services below**  Please tick NO or YES. If you tick ‘yes’ for any of the services (these may have been provided face-to-face, by video or telephone), please give the number of times you have used the service in the LAST 12 MONTHS. This can be your best guess as the exact number may be difficult to remember.  *The example shows: two visits to the Dentist in the last 12 months* | | | |
| --- | --- | --- | --- |
| **Over the LAST 12 MONTHS, have you used any of the following NHS Services?** | **No** | **Yes** | **Number of Appointments (Face-to-Face, telephone or video)** |
| *Example: Dentist* | □ | 🗹 | **2** |
| GP/ Family Doctor | □ | □ |  |
| Nurse | □ | □ |  |
| Outpatient appointments | □ | □ |  |
|  |  |  |  |
|  | **No** | **Yes** | **Number of Visits** |
| Emergency Care (casualty/ A&E) | □ | □ |  |
|  |  |  |  |
| Inpatient stay | **No** | **Yes** | **Number of days spent in hospital** |
|  | □ | □ |  |

|  | | |
| --- | --- | --- |
|  |  |  |
|  |  |  |
|  |  |  |
|  |  |  |
|  |  |  |
|  |  |  |
|  |  |  |
|  |  |  |

| Below are activities or services that may be paid for or subsidised by the NHS or Local Authority, Have you used any of these in the last 12 months (even if only once)? | | |
| --- | --- | --- |
| Local Authority or NHS provided service: | Yes | No |
| Attended a subsidised (e.g. vouchers) or paid for group-based programme such as Weight Watchers or Slimming World or similar meetings | □ | □ |
| Attended a subsidised or paid for gym, leisure centre or local sport facility to swim or take part in other physical activity sessions? | □ | □ |
| Attended a subsidised or paid for health trainer programme | □ | □ |
| Attended a subsidised or paid for exercise referral scheme | □ | □ |
| Attended a subsidised or paid for weight management programme at a Community Pharmacy | □ | □ |
| Have taken weight loss pills prescribed by the GP or hospital prescribed | □ | □ |
| Have had daily weight loss injections prescribed by the GP or hospital prescribed | □ | □ |
| Have had weekly weight loss injections prescribed by the GP or hospital prescribed | □ | □ |
| Have taken meal replacement drinks for weight loss prescribed by the GP or hospital prescribed where the NHS has paid for them (e.g. Optifast, Slim-Fast, The Cambridge Diet) | □ | □ |
| Attended an appointment with an NHS dietician for weight management | □ | □ |
| Used any other NHS or Local Authority provided weight management method  Please tell us: ______________________ | □ | □ |

**EQ-5D-5L**

| **DESCRIBING YOUR OWN HEALTH TODAY** | | | | |
| --- | --- | --- | --- | --- |
| Under each heading, please tick the ONE box that best describes your health TODAY | | | | |
| **Mobility** | I have no problems in walking about |  | 🞏 |  |
|  | I have slight problems in walking about |  | 🞏 |  |
|  | I have moderate problems in walking about |  | 🞏 |  |
|  | I have severe problems in walking about |  | 🞏 |  |
|  | I am unable to walk about |  | 🞏 |  |
|  |  |  | | |
| **Self-care** | I have no problems washing or dressing myself |  | 🞏 |  |
|  | I have slight problems washing or dressing myself |  | 🞏 |  |
|  | I have moderate problems washing or dressing myself |  | 🞏 |  |
|  | I have severe problems washing or dressing myself |  | 🞏 |  |
|  | I am unable to wash or dress myself |  | 🞏 |  |
|  |  |  | | |
| **Usual activities** | I have no problems doing my usual activities |  | 🞏 |  |
| *(e.g. work, study, housework, family or leisure activities)* | I have slight problems doing my usual activities |  | 🞏 |  |
|  | I have moderate problems doing my usual activities |  | 🞏 |  |
|  | I have severe problems doing my usual activities |  | 🞏 |  |
|  | I am unable to do my usual activities |  | 🞏 |  |
|  |  |  | | |
| **Pain/Discomfort** | I have no pain or discomfort |  | 🞏 |  |
|  | I have slight pain or discomfort |  | 🞏 |  |
|  | I have moderate pain or discomfort |  | 🞏 |  |
|  | I have severe pain or discomfort |  | 🞏 |  |
|  | I have extreme pain or discomfort |  | 🞏 |  |
|  |  |  |  |  |
| **Anxiety/Depression** | I am not anxious or depressed |  | 🞏 |  |
|  | I am slightly anxious or depressed |  | 🞏 |  |
|  | I am moderately anxious or depressed |  | 🞏 |  |
|  | I am severely anxious or depressed |  | 🞏 |  |
|  | I am extremely anxious or depressed |  | 🞏 |  |

9 0

8 0

7 0

6 0

5 0

4 0

3 0

2 0

1 0

100

The worst health you can imagine

0

The best health you can imagine

# **We would like to know how good or bad your health is TODAY.**

- This scale is numbered from 0 to 100.
- 100 means the best health you can imagine.

0 means the worst health you can imagine.

- Mark an X on the scale to indicate how your health is TODAY.
- Now, please write the number you marked on the scale in the box below.

**YOUR HEALTH TODAY** =

(UK (English) v.2 © 2009 EuroQol Group. EQ-5D™ is a trademark of the EuroQol Group)

**DUMMY TABLES**

**Table 1. Intervention costs**

|  | Units | Unit cost | Total costs |
| --- | --- | --- | --- |
| **Fixed cost** |  |  |  |
|  |  |  |  |
|  |  |  |  |
| **Variable cost (cost per person)** |  |  |  |
|  |  |  |  |
|  |  |  |  |
| **Weighing participants** |  |  |  |
|  |  |  |  |
|  |  |  |  |
| **Incentives for SMS+I group** |  |  |  |
|  |  |  |  |
|  |  |  |  |

**Table 2. Unit costs**

|  | **Unit cost (£)** | **Source** | **Notes** |
| --- | --- | --- | --- |
| GP appointments |  |  |  |
| Nurse appointments |  |  |  |
| A&E attendance |  |  |  |
| Outpatient appointments |  |  |  |
| Inpatient stay |  |  |  |

**Table 3. Healthcare use by time point**

|  |  | -3 to 0 months | | | 0-12 months | | | 12-24 months | | |
| --- | --- | --- | --- | --- | --- | --- | --- | --- | --- | --- |
|  |  | SMS + I | SMS only | Control | SMS + I | SMS only | Control | SMS + I | SMS | Control |
| GP appointments | N, M, SD |  |  |  |  |  |  |  |  |  |
| Nurse appointments | N, M, SD |  |  |  |  |  |  |  |  |  |
| A&E attendances | N, M, SD |  |  |  |  |  |  |  |  |  |
| Outpatient appointments | N, M, SD |  |  |  |  |  |  |  |  |  |
| Inpatient stays (days) | N, M, SD |  |  |  |  |  |  |  |  |  |
| **Health care use cost** | N, M, SD |  |  |  |  |  |  |  |  |  |

**Table 4: The EQ-5D tariff score and QALYs estimated using area under the curve**

|  |  | SMS + I | SMS only | Control |
| --- | --- | --- | --- | --- |
| EQ-5D utility score |  |  |  |  |
| Baseline | N, M, SD |  |  |  |
| 12 months | N, M, SD |  |  |  |
| 24 months | N, M, SD |  |  |  |
| **QALYs** | N, M, SD |  |  |  |

**Table 5. Incremental Cost Effectiveness Ratio**

|  |  | SMS + I vs Control | SMS only vs Control | SMS + I versus SMS only |
| --- | --- | --- | --- | --- |
| Difference in QALYs (unadjusted) | N, M, CI |  |  |  |
| Difference in QALYs (adjusted) | N, M, CI |  |  |  |
| Difference in Cost (unadjusted) | N, M, CI |  |  |  |
| Difference in Cost (adjusted) | N, M, CI |  |  |  |
| Difference in % weight loss (unadjusted) | N, M, CI |  |  |  |
| Difference in % weight loss (adjusted) | N, M, CI |  |  |  |
| **Incremental cost per QALY** |  |  |  |  |
| Unadjusted | N, M, CI |  |  |  |
| Adjusted | N, M, CI |  |  |  |
| **Incremental cost per % weight loss** |  |  |  |  |
| Unadjusted | N, M, CI |  |  |  |
| Adjusted | N, M, CI |  |  |  |

**Table 6. Lifetime cost and QALYs**

|  |  |  |  | Mean difference (95% CI) | | |
| --- | --- | --- | --- | --- | --- | --- |
|  | SMS + I | SMS only | Control | SMS + I vs Control | SMS only vs Control | SMS + I versus SMS only |
| Life years |  |  |  |  |  |  |
| QALYs |  |  |  |  |  |  |
| Total costs |  |  |  |  |  |  |
| Intervention costs (£) |  |  |  |  |  |  |
| NHS disease costs (£) |  |  |  |  |  |  |
| ICER (£ per QALY) |  |  |  |  |  |  |

**Table 7. Use of specific NHS or Local Authority funded weight loss services**

|  |  | SMS + I | SMS only | Control |
| --- | --- | --- | --- | --- |
| Attended a subsidised (e.g. vouchers) or paid for group-based programme such as Weight Watchers or Slimming World or similar meetings | N, n, % |  |  |  |
| Attended a subsidised or paid for gym, leisure centre or local sport facility to swim or take part in other physical activity sessions? | N, n, % |  |  |  |
| Attended a subsidised or paid for health trainer programme | N, n, % |  |  |  |
| Attended a subsidised or paid for exercise referral scheme | N, n, % |  |  |  |
| Attended a subsidised or paid for weight management programme at a Community Pharmacy | N, n, % |  |  |  |
| Have taken weight loss pills prescribed by the GP or hospital prescribed | N, n, % |  |  |  |
| Have had daily weight loss injections prescribed by the GP or hospital prescribed | N, n, % |  |  |  |
| Have had weekly weight loss injections prescribed by the GP or hospital prescribed | N, n, % |  |  |  |
| Have taken meal replacement drinks for weight loss prescribed by the GP or hospital prescribed where the NHS has paid for them (e.g. Optifast, Slim-Fast, The Cambridge Diet) | N, n, % |  |  |  |
| Attended an appointment with an NHS dietician for weight management | N, n, % |  |  |  |
| Used any other NHS or Local Authority provided weight management method | N, n, % |  |  |  |
| Other | N, n, % |  |  |  |
