# Supplementary material for: Text messaging with or without financial incentives versus a waitlist control for weight loss in men: cost-effectiveness analysis of the Game of Stones randomised controlled trial
Source: Lancet Reg Health Eur. 2025 May 21;54:101328. doi: 10.1016/j.lanepe.2025.101328 (PMC12149650; doi:10.1016/j.lanepe.2025.101328)
Supplement: Supplementary Figs. S1–S9 and Tables S1–S15 [file mmc2.docx]

Contents

[**1. Input materials** 2](#_Toc184952273)

[**2. Within trial analyses economic outcomes** 4](#_Toc184952274)

[**3. Long term modelling economic outcomes** 15](#_Toc184952275)

**4.** [**References** 27](#_Toc184952276)

**Supplementary Materials**

**Cost-effectiveness of text messages with loss-framed financial incentives for men with obesity: The Game of Stone trial**

# **1. Input materials**

**Supplementary Table 1: Unit costs for NHS resource use**

|  | **Unit cost (£)** | **Source** | **Notes** |
| --- | --- | --- | --- |
| GP appointments | 42 | PSSRU, 2022^1^ |  |
| Nurse appointments | 14.5 | Calculated | Calculated based on £52 nurse appointments per hr (PSSUR, 2022)^1^ and 15-minute contact duration (PSSUR, 2015)^2^ |
| ^a^A&E attendance | 278 | National Cost Collection 2021/2022^3^ |  |
| ^b^Outpatient appointments | 162 | National Cost Collection 2021/2022^3^ |  |
| Inpatient stay | 850 | Public Health Scotland 2019/2020^4^ | Inflated to 2021/2022 using consumer price index for health care |

^a^ weighted average of all emergency medicine episodes except those for which no investigation or significant treatment was required or the patient was dead on arrival. ^b^ weighted average of all consultant led and non-consultant-led outpatient attendances.

**Supplementary Table 2: Model inputs**

| **Inputs** | **Source** |
| --- | --- |
| UK Population numbers by single year of age (0-100) and sex | Human Mortality Database, 2021^5^ |
| Population mortality rates by single year of age and sex | Human Mortality Database, 2020^5^ |
| BMI distribution by single year of age and sex | Kent et al 2019 ^6^  (Based on the Health Survey of England) |
| Disease incidence and baseline prevalence by single year of age and sex | GBD Results Tool^7^ |
| Disease case fatality rate by single year of age and sex |  |
| Background trends in disease incidence and case-fatality rates |  |
| Relative risks (This gives the effect of changes in BMI on disease incidence and total mortality)^a^  **Disease**  **Age start** **Age end** **rr_mean** **rr_se**  hid 0 59 1.5 0.039  ihd 60 69 1.4 0.031  ihd 70 79 1.31 0.033  ihd 80 100 1.3 0.055  stroke 0 59 1.76 0.075  stroke 60 69 1.49 0.056  stroke 70 79 1.33 0.056  stroke 80 100 1.1 0.083  diabetes 0 100 2.16 0.067  cirrhosis 0 100 1.79 0.077  cancercol 0 100 1.24 0.016  cancerki 0 100 1.24 0.039  cancerLiver 0 100 1.47 0.078  cancerpanc 0 100 1.1 0.016  mortality 0 49 1.61 0.0125  mortality 50 69 1.43 0.03  mortality 70 100 1.21 0.035  Where hid= ischemic heart disease; cancercol = colorectal cancer; cancerki= kidney cancer; cancerpanc= pancreatic cancer; rr =relative risk, se= standard  error | Kent et al, 2019^6^  (Model input which is freely available at [GitHub - seamuskent/PRIMEtime-CE-Obesity](https://github.com/seamuskent/PRIMEtime-CE-Obesity) |
| Disease-specific and background utility weights | Sullivan et al, 2011^8^  Details in Supplementary Table 3 |
| Disease costs | Kent et al, 2019^6^  Detailed in Supplementary Table 3 |
| Weight loss at the end of 12 months | Based on the GOS trial data (This study) |
| Standard error for the treatment effect on weight loss difference at 12 months between text messages alone vs. control and text messages with financial incentives vs. control. These were calculated as 0.92 and 0.89 respectively^b^  The mean treatment effect on weight loss difference at 12 months between text messages alone vs. control = -1.37 (95% CI, -3.18, 0.44)  The mean treatment effect on weight loss difference at 12 months between text messages with financial incentives vs. control = -3.6 (95% CI, -5.34, -1.86)  Assuming a normal distribution, the standard errors were calculated using the formula (UB-LB)/ (1.96*2)  Where UB and LB= upper bound and lower bound of the CI. | Based on the GOS trial data (This study) |
| Rate of weight regain at 24 months:  Text messages alone = 0.37 Kg/year  Text messages with financial incentives = 1.18 Kg/year  Control= 0.3Kg/year | Text messages alone and text messages with financial incentives based on the GOS trial data (This study)  Control based on Hartmann-Boyce et al 2022^9^ |
| Intervention cost | Based on the GOS trial data (This study) |

^a^ log-normal distribution was assumed in the probabilistic sensitivity analysis (PSA). ^b^ normal distribution was assumed in the probabilistic sensitivity analysis (PSA). GBD= Global Health Data Exchange

**Supplementary Table 3: Utility weights and unit disease costs**

| **Disease-specific disutility weights, estimated for the UK (Sullivan et al, 2011**^8^ **(**(Web Table 5) | | |
| --- | --- | --- |
| **Diseases** | **Mean utility (SE)^*^** | **ICD9 code** |
| Ischaemic heart disease incidence | -0.063 (0.013) | icd410 |
| Ischaemic heart disease prevalence | -0.037 (0.026) | icd412 |
| stroke incidence | -0.117 (0.012) | icd436 |
| stroke prevalence | -0.073 (0.024) | icd438 |
| Diabetes | -0.071 (0.005) | icd250 |
| Colon and rectum cancer | -0.067 (0.017) | icd153 |
| Liver cancer | -0.093 (0.044) | icd155 |
| Kidney cancer | -0.048 (0.041) | icd189 |
| Pancreatic cancer | -0.010 (0.026) | icd202 |
| **Background utility weight estimated for the UK** | | |
| **Age in years** | **Mean utility (SE)** | **Source** |
| 10-19 | 0.913 (0.0045) | Sullivan et al, 2011^8^  (Web Table 1) |
| 20-29 | 0.905 (0.0021) |  |
| 30-39 | 0.879 (0.0021) |  |
| 40-49 | 0.837 (0.0028) |  |
| 50-59 | 0.798 (0.0035) |  |
| 60-69 | 0.774 (0.0039) |  |
| 70-79 | 0.723 (0.0049) |  |
| 80+ | 0.657 (0.0075) |  |
| **Disease costs** | | |
| **Disease** | **Mean cost (2014) in £ (SE= assumed 10% of the mean)** | **Source** |
| Ischaemic heart disease | 1,905 | ^10^Kent et al, 2019^6^  (Inflated to 2021/2022 using the UK consumer price index for healthcare. |
| Stroke | 843 |  |
| Diabetes | 444 |  |
| Colon and rectum cancer | 809 |  |
| Liver cancer | 1,532 |  |
| Kidney cancer | 618 |  |
| Pancreatic cancer | 3,074 |  |

^*^SE: standard error; Note: normal and gamma distributions were assumed for utility and cost, respectively, in the PSA. The 10% assumption for the SE, in the model, was based on Blakely et al^10^. The parameters for the gamma distribution were calculated using the formula from Briggs et al.^11^ : α =Mean^2^/SE^2^ and β= SE^2^ /Mean

# **2. Within trial analyses economic outcomes**

**Supplementary Table 4: Healthcare use by time point: mean, SD, number (observed data)**

|  | **Baseline** | | | **0-24 months (2 years)** | | |
| --- | --- | --- | --- | --- | --- | --- |
| **Variables: mean, (SD), n** | **Text messages with financial incentives N=196** | **Text message alone N=194** | **Control**  **N=195** | **Text messages with financial incentives N=196** | **Text message alone N=194** | **Control**  **N=195** |
| GP appointments | 1.19 (1.52); 188 | 1.38 (1.88); 190 | 1.44 (2.47);191 | 4.79 (6.18); 113 | 5.51 (8.48); 96 | 4.59 (4.73); 117 |
| Nurse appointments | 0.61 (1.05); 179 | 0.74 (1.21); 186 | 0.76 (1.44); 185 | 5.42 (20.0); 115 | 3.32 (3.98); 97 | 3.16 (4.72); 113 |
| A&E attendances | 0.10 (0.41); 182 | 0.13 (0.45); 191 | 0.13 (.47); 186 | 0.45 (0.88); 115 | 0.67 (1.32); 99 | 0.49 (0.98); 121 |
| Outpatient appointments | 0.38 (0.86); 182 | 0.63 (1.39); 187 | 0.61 (1.41); 184 | 2.96 (6.10);112 | 2.68 (4.55); 98 | 2.33 (3.23); 118 |
| Inpatient stays (days) | 0.16 (1.12); 183 | 0.18 (1.06); 190 | 0.027 (.243); 184 | 0.91 (3.48); 115 | 1.36(4.43); 98 | 0.96 (3.43); 123 |
| Healthcare use cost | 245 (1035);171 | 361 (1081);179 | 204 (385);172 | 1356 (3526); 99 | 1639 (3350); 83 | 1526 (3523); 105 |

**Supplementary Table 5: The EQ-5D tariff score and QALYs estimated using the area under the curve: mean SD, number (observed 2 years data)**

| **Variables: mean, (SD), n** | **Text messages with financial incentives (N= 196)** | **Text message alone (N=194)** | **Control (N=195)** |
| --- | --- | --- | --- |
| **EQ-5D utility score** |  |  |  |
| Baseline | 0.726 (0.229); 194 | 0.741 (0.230); 192 | 0.721 (0.207); 191 |
| 12 months | 0.762 (0.240); 143 | 0.719 (0.258); 127 | 0.764 (0.203); 148 |
| 24 months | 0.732 (0.243); 125 | 0.748 (0.215); 101 | 0.787 (0.185); 121 |
| **QALYs at 2 years** | 1.46 (0.43); 120 | 1.47 (0.74); 93 | 1.52 (0.32); 112 |

**Supplementary Table 6 Adjusted mean costs and QALYs at 2 years across intervention arms (from multiple imputed 2 years data)**

|  | **Mean total costs in £**  **(95% CI)** | **^#^Mean intervention costs in £** | **Mean health care costs in £** | **Mean QALYs** |
| --- | --- | --- | --- | --- |
| Control | 1,531 (1,064; 1,998) | 0 (0; 0) | 1,531 (1,056; 2073) | 1.49 (1.46; 1.52) |
| Text message alone | 1,930 (1,298; 2,561) | 110 (98; 121) | 1,820 (1,189; 2474) | 1.42 (1.38; 1.45) |
| Text messages with financial incentives | 1,660 (1,154; 2,166) | 243 (219; 267) | 1,417 (919; 1,855) | 1.47 (1.45; 1.54) |

# Intervention cost is calculated at 12 months

**Supplementary Table 7a. Incremental Cost Effectiveness Ratio from GLM^**^ (pairwise comparison using imputed 2 years data) ^#^**

|  | **Text messages with financial incentives vs Control** | **Text message alone vs Control** | **Text messages with financial incentives vs Text messages alone** |
| --- | --- | --- | --- |
| Difference in QALYs (unadjusted) | -0.014 (95% CI: -0.097; 0.070)  (*P*= 0.748) | -0.043 (95% CI: -0.127; 0.041)   (*P=* 0.314) | 0.029 (95% CI: -0.055; 0.114)  (*P*= 0.495) |
| Difference in QALYs (adjusted)^a^ | -0.019 (-0. 065; 0.027)  (*P*=0.408) | -0. 071 (-0.119; -0.024)  *(P*= 0.003) | 0.052 (0.004; 0.099)  (*P*= 0.033) |
| Difference in Cost (unadjusted) | -121 (-795.7; 554)  (*P* = 0.724) | 272 (-582; 1,127)  (*P*= 0.531) | -394 (-1,174; 387)  (*P*= (0.322) |
| Difference in Cost (adjusted)^b^ | 129 (-495; 753)  (*P*= 0.685) | 399 (-340; 1,137)  (*P*= (0.289) | -270 (-952; 413)  (*P*= (0.438) |
| Difference in % weight loss at 12 Months (unadjusted) | 3.26 (1.93; 4.60)  (*P*=0.001) | 1.46 (0. 02; 2.90)  (*P*= 0.046) | 1.80 (0.37; 3.23)  (*P*=0.014) |
| Difference in % weight loss at 12 Months (adjusted)^c^ | 3.23 (1.89; 4.57)  (*P*=0.001) | 1.42 (-0. 02; 2.87)  (*P*=0.053) | 1.81 (0.38; 3.24)  (*P*=0.014) |
| Difference in % weight loss at 24 Months (unadjusted) | 1.05 (-0.58; 2.68)  (*P*=0.207) | 0.09 (-1.61; 1.79)  (*P*=0.915) | 0.96 (-0.63; 2.54)  (*P*=0.237) |
| Difference in % weight loss at 24 Months (adjusted)^c^ | 1.02 (-0.61; 2.65)  (*P*= 0.218) | 0.06 (-1.64; 1.76)  (*P*= 0.944) | 0.96 (-0.62; 2.54)  (*P*= 0.234) |
| **Incremental cost per QALY** |  |  |  |
| Unadjusted | 8,643 (-60,603; 41,484) | Dominated (-6,325) | Dominant (-13,552) |
| Adjusted | Dominated (-6,789) | Dominated (-5,620) | Dominant (-5,192) |
| **Incremental cost per % weight loss_12M** |  |  |  |
| Unadjusted | Dominant (-37) | 186 (-58; 790) | Dominant (-218) |
| Adjusted | 40 (-65; 219) | 281 (-84; 978) | Dominant (-149) |
| **Incremental cost per % weight loss_24M** |  |  |  |
| Unadjusted | Dominant (-115) | 3,022 (-7,215; 6,924) | Dominant (-409) |
| Adjusted | 126 (-899; 1,984) | 6,650 (-13,837;12,280) | Dominant (-281) |

#The full incremental analysis is presented in the next table (i.e. Supplementary Table 7b). ^a^ baseline utility index, study centre, and recruitment method were used for adjustment. ^b^ baseline total costs, study centre, and recruitment method were used for adjustment. ^c^ baseline weight, study centre, and recruitment method were used for adjustment. ^**^GLM= Generalised Linear Model

**Supplementary Table 7b. Full incremental analysis (Imputed 2 years data)**

|  | **Text messages with financial incentives vs Control** | **Text message alone vs Control** | **Text messages with financial incentives vs Text messages alone** |
| --- | --- | --- | --- |
| **Incremental cost per QALY** |  |  |  |
| Unadjusted | 8,643 (95% CI: -60,603; 41,484) | dominated | - |
| Adjusted | dominated | dominated | - |
| **Incremental cost per % weight loss_12M** |  |  |  |
| Unadjusted | dominant | dominated | - |
| Adjusted | 40 (-65; 219) | dominated | - |
| **Incremental cost per % weight loss_24M** |  |  |  |
| Unadjusted | Dominant | Dominated | - |
| Adjusted | 126 (-899; 1,984) | Dominated | - |

**Supplementary Figure 1a: Cost-effectiveness plane (Imputed 2 years data adjusted).** Baseline utility index, study centre, and recruitment method were used for adjusting QALYs. ^b^ baseline total costs, study centre, and recruitment method were used for adjusting costs.

**Supplementary Figure 1b: Cost-effectiveness acceptability curves (Imputed 2 years data adjusted).** Baseline utility index, study centre, and recruitment method were used for adjusting QALYs. ^b^ baseline total costs, study centre, and recruitment method were used for adjusting costs.

**Supplementary Table 8a: Incremental Cost Effectiveness Ratio from GLM^**^ (pairwise comparison using complete case 2 years data) ^#^**

|  | **Text messages with financial incentives vs Control** | **Text message alone vs Control** | **Text messages with financial incentives vs Text messages alone** |
| --- | --- | --- | --- |
| Difference in QALYs (unadjusted) | -0.0242 (95% CI: -0.1336; 0.0851)  (P=0.664) | -0.0071 (95% CI: -0.1243; 0.1101)  (P=0.906) | -0.0172 (95% CI: -0.1344; 0.10)  (*P*=0.774) |
| Difference in QALYs (adjusted)^a^ | -0.0099(-0.0649; 0.0449)  (*P*=0.722) | -0.0419 (-0.1008; 0.0169)  (*P*=0.163) | 0.0319 (-0.0271; 0.0909)  (*P*=0.289) |
| Difference in Cost (unadjusted) | 13 (-762; 788)  (*P*=0.974) | -36 (-849; 777)  (*P*=0.930) | 49 (-767; 866)  (*P*=0.905) |
| Difference in Cost (adjusted)^b^ | 314 (-676; 1305)  (*P*=0.534) | 175 (-1099; 1449)  (*P*=0.788) | 139 (-624; 902)  (*P*=0.721) |
| Difference in % weight loss at 12 Months (unadjusted) | 3.51 (1.89; 5.13)  (*P*=0.0001) | 1.54 (-0 .1; 3.18)  (*P*=0.065) | 1.97 (0.32; 3.61)  (*P*=0.019) |
| Difference in % weight loss at 12 Months (adjusted)^c^ | 3.50 (1.87; 5.10)  (*P=*0.001) | 1.59 (-0.06; 3.23)  (*P*=0.059) | 1.92 (0.27; 3.56)  (*P*=0.022) |
| Difference in % weight loss at 24 Months (unadjusted) | 2.09 (- 0.05; 4.23)  (*P*=0.056) | 0.53 (-1.77; 2.82)  (*P*=0.653) | 1.57 (-0.72; 3.85)  (*P*=0.264) |
| Difference in % weight loss at 24 Months (adjusted)^c^ | 1.93 (-0.02; 4.09)  (*P*=0.079) | 0.62 (-1.68; 2.92)  (*P*=0.599) | 1.32 (-0.99; 3.62)  (*P*=0.180) |
| **Incremental cost per QALY** |  |  |  |
| Unadjusted | Dominated (-537) | 5,070 (-112,622; 111,789) | Dominated (-2,866) |
| Adjusted | Dominated (-31,717) | Dominated (-4,177) | 4,344 ( -147,454; 108,839) |
| **Incremental cost per % weight loss_12M** |  |  |  |
| Unadjusted | 3.7 (-263; 89) | Dominant (-23) | 25 (-594; 167) |
| Adjusted | 90 (-78; 380) | 110 (86; 133) | 73 (-760; 1,021) |
| **Incremental cost per % weight loss_24M** |  |  |  |
| Unadjusted | 6.2 (-799; 756) | Dominant (-68) | 31 (-1,500; 2,074) |
| Adjusted | 163 (-559; 1,336 ) | 282 (-4,096; 5,043) | 106 (-3,049; 4,438) |

#The full incremental analysis is presented in the next table (i.e. Supplementary Table 8b). ^a^ baseline utility index, study centre, and recruitment method were used for adjustment. ^b^ baseline total costs, study centre, and recruitment method were used for adjustment. ^c^ baseline weight, study centre, and recruitment method were used for adjustment. ^**^GLM= Generalised Linear Model

**Supplementary Table 8b: Full incremental analysis (complete case 2 years data)**

|  | **Text messages with financial incentives vs Control** | **Text message alone vs Control** | **Text messages with financial incentives vs Text messages alone** |
| --- | --- | --- | --- |
| **Incremental cost per QALY** |  |  |  |
| Unadjusted | dominated | 5,070 (-112,622; 111,789) | - |
| Adjusted | dominated | dominated | - |
| **Incremental cost per % weight loss_12M** |  |  |  |
| Unadjusted | - | Dominant | Extendedly dominated |
| Adjusted | 90 (-78; 380) | Extendedly dominated | - |
| **Incremental cost per % weight loss_24M** |  |  |  |
| Unadjusted | - | Dominant | Extendedly dominated |
| Adjusted | 163 (-559; 1,336) | Extendedly dominated | - |

**Supplementary Figure 2a: Cost-effectiveness plane (complete case_2 years data adjusted).**

**Supplementary Figure 2b: Cost-effectiveness acceptability curves (complete case 2 years data adjusted)**

**Supplementary Table 9: Number of completers (participants present both at baseline, 12M, and 24 months); (%missing)**

|  | **Text messages with financial incentives (N=196)** | **Text messages alone (N=194)** | **Control (N=195)** |
| --- | --- | --- | --- |
| EQ5D | 120 (39%) | 93 (52%) | 112 (42.5%) |
| QALY | 120 (39%) | 93 (52%) | 112 (42.5%) |
| Total health care visit | 89 (55%) | 78 (60%) | 96 (51%) |

**Supplementary Table 10: Missing data (resource use and EQ5D)**

|  | **Baseline** | | | **0-24 months** | | |
| --- | --- | --- | --- | --- | --- | --- |
| **Variables: n (%)** | **Text messages with financial incentives N=196** | **Text messages alone**  **N=194** | **Control**  **N=195** | **Text messages with financial incentives N=196** | **Text messages alone**  **N=194** | **Control**  **N=195** |
| GP appointments | 8 (4%) | 4 (2%) | 4 (2%) | 83 (42%) | 98 (51%) | 78 (40%) |
| Nurse appointments | 17 (9%) | 8 (4%) | 10 (5%) | 81 (41%) | 97 (50%) | 82 (42%) |
| A&E attendances | 14 (7%) | 3 (1.5%) | 9 (5%) | 81 (41%) | 95 (49%) | 74 (38%) |
| Outpatient appointments | 14 (7%) | 7 (4%) | 11 (6%) | 84 (43%) | 96 (49%) | 77 (39%) |
| Inpatient stays (days) | 13 (7%) | 4 (2%) | 11 (6%) | 81 (41%) | 96 (49%) | 72 (37%) |
| Health care use cost | 25 (13%) | 15 (8%) | 23 (12%) | 97 (49%) | 111 (57%) | 90 (46%) |
| EQ-5D utility score | 2 (1%) | 2 (1%) | 4 (2%) | 76 (39%) | 101 (52%) | 80 (41%) |

# **2. Long term modelling economic outcomes**

**Supplementary Table 11a. Health benefits and health care costs over lifetime for text messages with financial incentives and text messages alone vs waitlist control** (**Base case**)**.** Note: Both costs and QALYs are discounted at a rate of 3.5% per annum.

|  | **Control** | **Text messages with financial incentives** | **Difference** (95% uncertainty) |
| --- | --- | --- | --- |
|  |  |  |  |
| Life-years | 16.79 | 16.81 | 0.02 (0.012; 0.025) |
| QALYs | 12.47 | 12.48 | 0.02 (0.007; 0.029) |
| **Total costs (£)** | 15,101 | 15,277 | 176 (43; 311) |
| Treatment costs (£) | 0 | 243 | 243(243; 243) |
| NHS disease costs (£) | 15,101 | 15,034 | -66.6 (-200; 68) |
| **^p^**ICER (£ per QALY) |  |  | 9,748 (7,705; 11,791) |
| **Disease incidence (per 100,000 persons)** |  |  |  |
| Ischemic heart disease. | 67,690 | 67,485 | -205 (-314; -93) |
| Stroke | 19,406 | 19,363 | -43 (-120; 34) |
| Type 2 diabetes | 28,057 | 27,689 | -368 (-421; -306) |
| Cancer^#^ | 32,516 | 32,505 | -11 (-75; 54) |
|  | | | |
|  | **Control** | **Text messages alone** | **Difference** (95% uncertainty)**)** |
| Life-years | 16.79 | 16.82 | 0.03 (0.021; 0.033) |
| QALYs | 12.47 | 12.49 | 0.03 (0.015; 0.037) |
| **Total costs (£)** | 15,101 | 15,117 | 16.5 (-117; 152) |
| Treatment costs (£) | 0 | 110 | 110 (110; 110) |
| NHS disease costs (£) | 15,101 | 15,007 | -94 (-227; 42) |
| **^P^**ICER (£ per QALY) |  |  | 628 (-5,914; 5,384) |
| **Disease incidence (per 100,000 persons** |  |  |  |
| Ischemic heart disease | 67,690 | 67,359 | -331 (-439; -217) |
| Stroke | 19,406 | 19,341 | -66 (-142; 12) |
| Type 2 diabetes | 28,057 | 27,435 | -625 (-674; -552) |
| Cancer^#^ | 32,516 | 32,492 | -24 (-88; 41) |

^P^ICER from the pairwise comparison (i.e. text messages with financial incentives vs control and text messages alone vs control. ^#^ Includes cancers of the colon, liver, kidney, and pancreas. Note: Some numbers are rounded. The full incremental analysis is presented in the next table (i.e. supplementary Table 11b).

**Supplementary Table 11b: Base case results of cost-effectiveness analyses from full incremental analysis**

|  | **Incremental QALY** | **Incremental Cost (£)** | **ICER** |
| --- | --- | --- | --- |
| Control | 0 | 0 | - |
| Text messages alone | 0.026 | 17 | 628 |
| Text messages with financial incentives | 0.018 | 176 | Dominated |

Note: Some numbers are rounded

**
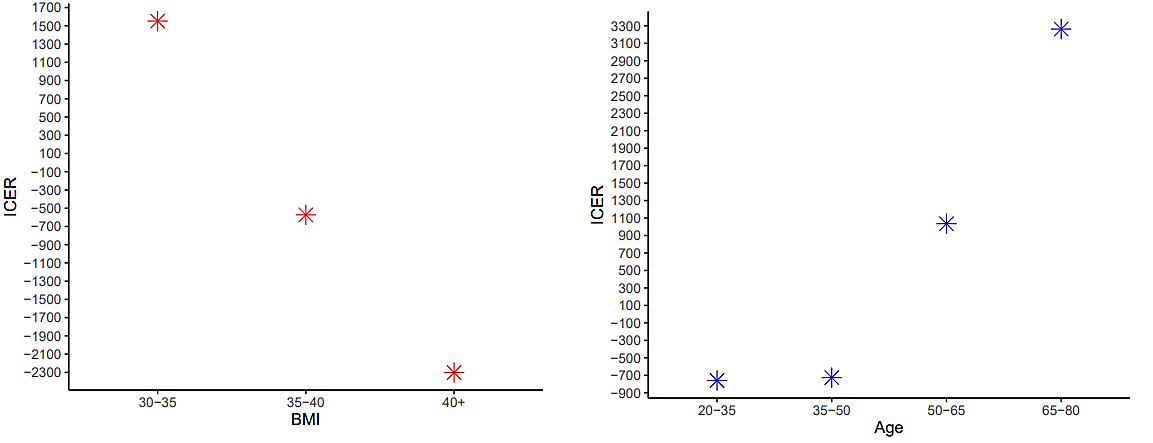
**

**Supplementary Figure. 3. Incremental cost-effectiveness ratio by BMI and age categories**. In all cases, the text message alone strategy remains the optimal strategy based on full incremental analysis.

**Supplementary Figure 4a: Cost-effectiveness plane**

**Supplementary Figure 4b: Cost-effectiveness acceptability curves**.

**Supplementary Table 12a:** **Health benefits and health care costs over lifetime for text messages with financial incentives and text messages alone vs waitlist control** **(assuming equal weight regain time; 5 years).** Note: Both costs and QALYs are discounted at a rate of 3.5% per annum

|  | **Control** | **Text messages with financial incentives** | Difference (95% uncertainty) |
| --- | --- | --- | --- |
|  |  |  |  |
| Life-years | 16.78 | 16.80 | 0.026 (0.02; 0.033) |
| QALYs | 12.46 | 12.48 | 0.026 (0.015; 0.037) |
| **Total costs (£)** | 15,126 | 15,274 | 148 (14; 284) |
| Treatment costs (£) | 0 | 243 | 243 (243; 243) |
| NHS disease costs (£) | 15,126 | 15,031 | -95 (-229; 41) |
| **^p^**ICER (£ per QALY) |  |  | 5,721 (5,434; 6,007) |
| **Disease incidence (per 100,000 persons)** |  |  |  |
| Ischemic heart disease | 67,780 | 67,476 | -304 (-413; -192) |
| Stroke | 19,424 | 19,361 | -63 (-139; 15) |
| Type 2 diabetes | 28,232 | 27,671 | -561 (-613; -498) |
| Cancer^#^ | 32,522 | 32,504 | -18 (-83; 46) |
|  | | | |
|  | **Control** | **Text messages alone** | **Difference** (95% uncertainty) |
| Life-years | 16.78 | 16.79 | 0.010 (0.004; 0.017) |
| QALYs | 12.46 | 12.47 | 0.010 (0; 0.021) |
| Total costs (£) | 15,126 | 15,198 | 72 (-62; 208) |
| Treatment costs (£) | 0 | 110 | 110 (110; 110) |
| NHS disease costs (£) | 15,126 | 15,088 | -38 (-172;98) |
| **^P^**ICER (£ per QALY) |  |  | 7,078 (-7,002; 22,059) |
| Disease incidence (per 100,000 persons |  |  |  |
| Ischemic heart disease | 67,780 | 67,661 | -120 (-229; -7) |
| Stroke | 19,424 | 19,399 | -25 (-102; 53) |
| Type 2 diabetes | 28,232 | 28,006 | -226 (-281; -162) |
| Cancer^#^ | 32,522 | 32,515 | -7 (-72; 58) |

^P^ICER from the pairwise comparison (i.e. text messages with financial incentives vs control and text messages alone vs control. ^#^ Includes cancers of the colon, liver, kidney, and pancreas. Note: Some numbers are rounded. The full incremental analysis is presented in the next table (i.e. supplementary Table 12b)

**Supplementary Table 12b: Incremental cost-effectiveness analyses result (full incremental analysis)**

|  | **Incremental QALY** | **Incremental Cost (£)** | **ICER** |
| --- | --- | --- | --- |
| Control | 0 | 0 | - |
| Text messages alone | 0.010 | 72 | Extendedly dominated |
| Text messages with financial incentives | 0.026 | 148 | 4,836 |

Some numbers are rounded

**Supplementary Figure 5a: Cost-effectiveness plane**

**Supplementary Figure 5b: Cost-effectiveness acceptability curves**

**Supplementary Table 13a. Health benefits and health care costs over lifetime for text messages with financial incentives and text messages alone vs waitlist control**(**assuming 10% of weight loss at 12 months maintained beyond 5 years**). Note: Both costs and QALYs are discounted at a rate of 3.5% per annum

|  | **Control** | **Text messages with financial incentives** | **Difference** (95% uncertainty) |
| --- | --- | --- | --- |
|  |  |  |  |
| Life-years | 16.78 | 16.85 | 0.067 (0 .061; 0.073) |
| QALYs | 12.46 | 12.52 | 0.061 (0.050; 0.072) |
| **Total costs (£)** | 15,126 | 15,185 | 59 (-74; 194) |
| Treatment costs (£) | 0 | 243 | 243 (243; 243) |
| NHS disease costs (£) | 15,126 | 14,942 | -184 (-347; -49) |
| **^p^**ICER (£ per QALY) |  |  | 973 (926; 1,020) |
| **Disease incidence (per 100,000 persons)** |  |  |  |
| Ischemic heart disease | 67,780 | 66,792 | -988 (-1,099; -885) |
| Stroke | 19,424 | 19,261 | -162 (-239; -89) |
| Type 2 diabetes | 28,232 | 26,722 | -1,510 (-1,564; -1,454) |
| Cancer^#^ | 32,522 | 32,360 | -163 (-224; -99) |
|  | | | |
|  | **Control** | **Text messages alone** | **Difference** (95% uncertainty) |
| Life-years | 16.78 | 16.82 | 0.035 (0.029; 0.041) |
| QALYs | 12.46 | 12.49 | 0.032 (0.020; 0.043) |
| Total costs (£) | 15,126 | 15,144 | 18 (-115;154) |
| Treatment costs (£) | 0 | 110 | 110 (110; 110) |
| NHS disease costs (£) | 15,126 | 15,034 | -92 (-225; 44) |
| **^P^**ICER (£ per QALY) |  |  | 555 (447; 663) |
| Disease incidence (per 100,000 persons |  |  |  |
| Ischemic heart disease | 67,780 | 67,240 | -541 (-643; -426) |
| Stroke | 19,424 | 19,338 | -86 (-162; -10) |
| Type 2 diabetes | 28,232 | 27,419 | -813 (-857; -743) |
| Cancer^#^ | 32,522 | 32,427 | -96 (-157; -30) |

^P^ICER from the pairwise comparison (i.e. text messages with financial incentives vs control and text messages alone vs control. ^#^ Includes cancers of the colon, liver, kidney, and pancreas. Note: Some numbers are rounded. The full incremental analysis is presented in the next table (i.e. supplementary Table 13b).

**Supplementary Table 13b: Incremental cost-effectiveness analyses result (full incremental analysis)**

|  | **Incremental QALY** | **Incremental Cost (£)** | **ICER** |
| --- | --- | --- | --- |
| Control | 0 | 0 | - |
| Text messages alone | 0.032 | 18 | 555 |
| Text messages with financial incentives | 0.061 | 59 | 1,429 |

Note: Some numbers are rounded

**Supplementary Figure 6a: Cost-effectiveness plane**

**Supplementary Figure 6b: Cost-effectiveness acceptability curves**

**Supplementary Table 14a: Health benefits and health care costs over lifetime for text messages with financial incentives and text messages alone vs waitlist control** (**Baseline weight carried forward method was applied to impute missing weight measurements unlike the base case where multiple imputation was used)**. Note: Both costs and QALYs are discounted at a rate of 3.5% per annum

|  | **Control** | **Text messages with financial incentives** | **Difference** (95% uncertainty) |
| --- | --- | --- | --- |
|  |  |  |  |
| Life-years | 16.77 | 16.79 | 0.017 (0.011; 0.023) |
| QALYs | 12.45 | 12.47 | 0.017 (0.006; 0.028) |
| **Total costs (£)** | 15,160 | 15,341 | 181 (46; 316) |
| Treatment costs (£) | 0 | 243 | 243 (243; 243) |
| NHS disease costs (£) | 15,160 | 15,098 | -62 (-197; 73) |
| **^p^**ICER (£ per QALY) |  |  | 10,709 (10,357; 11,061) |
| **Disease incidence (per 100,000 persons)** |  |  |  |
| Ischemic heart disease | 67,889 | 67,695 | -194 (-304; -82) |
| Stroke | 19,447 | 19,405 | -41 (-119; 36) |
| Type 2 diabetes | 28,446 | 28,078 | -367 (-424; -306) |
| Cancer^#^ | 32,529 | 32,518 | -10 (-75; 54) |
|  | | | |
|  | **Control** | **Text messages alone** | **Difference** (95% uncertainty) |
| Life-years | 16.77 | 16.78 | 0.01 (0.004; 0.017) |
| QALYs | 12.45 | 12.46 | 0.01( -0.001; 0.0211) |
| **Total costs (£)** | 15,160 | 15,233 | 72 (-62; 208) |
| Treatment costs (£) | 0 | 110 | 110 (110; 110) |
| NHS disease costs (£) | 15,160 | 15,113 | -38 |
| **^P^**ICER (£ per QALY) |  |  | 7,130 (-15,573; 42,062) |
| **Disease incidence (per 100,000 persons** |  |  |  |
| Ischemic heart disease | 67,889 | 67,768 | -121 (-231; -8) |
| Stroke | 19,447 | 19,422 | -25 (-102; 53) |
| Type 2 diabetes | 28,446 | 28,207 | -238 (-295; -175) |
| Cancer^#^ | 32,529 | 32,521 | -7.5 (-72; 57) |

^P^ICER from the pairwise comparison (i.e. text messages with financial incentives vs control and text messages alone vs control. ^#^ Includes cancers of the colon, liver, kidney, and pancreas. Note: Some numbers are rounded. The full incremental analysis is presented in the next table (i.e. supplementary Table 14b)

**Supplementary Table 14b: Incremental cost-effectiveness analyses result (full incremental analysis)**

|  | **Incremental QALY** | **Incremental Cost**  **(£)** | **ICER** |
| --- | --- | --- | --- |
| Control | 0 | 0 | - |
| Text messages alone | 0.01 | 72 | 7,130 |
| Text messages with financial incentives | 0.017 | 181 | 16,124 |

Note: Some numbers are rounded

**Supplementary Figure 7a: Cost-effectiveness plan**

**Supplementary Figure 7b:** Cost-effectiveness acceptability curves

**Supplementary Table 15a: Health benefits and health care costs over lifetime for text messages with financial incentives and text messages alone vs waitlist control. (Complete case weight measurement data were used to estimate weight loss** **and weight regain unlike the base case where missing data were imputed using multiple imputations)**. Note: Both costs and QALYs are discounted at a rate of 3.5% per annum

|  | **Control** | **Text messages with financial incentives** | **Difference** (95% uncertainty) |
| --- | --- | --- | --- |
|  |  |  |  |
| Life-years | 16.78 | 16.81 | 0.031 (0.025; 0.038) |
| QALYs | 12.46 | 12.49 | 0.031 (0.019; 0.045) |
| **Total costs (£)** | 15,132 | 15,261 | 130 (-4;265) |
| Treatment costs (£) | 0 | 243 | 243 (243; 243) |
| NHS disease costs (£) | 15,132 | 15,018 | -113 (-247; 22) |
| **^p^**ICER (£ per QALY) |  |  | 4,185(3,993; 4,377) |
| **Disease incidence (per 100,000 persons)** |  |  |  |
| Ischemic heart disease | 67,796 | 67,432 | -364 (-472; -251) |
| Stroke | 19,428 | 19,353 | -75 (-152; 2) |
| Type 2 diabetes | 28,262 | 27,593 | -669 (-721; -606) |
| Cancer^#^ | 32,523 | 32,501 | -23 (-86;43) |
|  | | | |
|  | **Control** | **Text messages alone** | **Difference** (95% uncertainty) |
| Life-years | 16.78 | 16.80 | 0.023 (0.016; 0.029) |
| QALYs | 12.46 | 12.48 | 0.022 (0.011; 0.033) |
| Total costs (£) | 15,132 | 15,161 | 29 (-105; 165) |
| Treatment costs (£) | 0 | 110 | 110 (110;110) |
| NHS disease costs (£) | 15,132 | 15,051 | -81 (-215;55) |
| **^P^**ICER (£ per QALY) |  |  | 1,291 (-219; 2,801) |
| Disease incidence (per 100,000 persons |  |  |  |
| Ischemic heart disease | 67,796 | 67,523 | -273 (-381; -158) |
| Stroke | 19,428 | 19,372 | -55 (-132; 22) |
| Type 2 diabetes | 28,262 | 27,743 | -520 (-572; -451) |
| Cancer^#^ | 32,523 | 32,505 | -18 (-82; 47) |

^P^ICER from the pairwise comparison (i.e. text messages with financial incentives vs control and text messages alone vs control. ^#^ Includes cancers of the colon, liver, kidney, and pancreas. Note: Some numbers are rounded. The full incremental analysis is presented in the next table (i.e. supplementary Table 15b).

**Supplementary Table 15b: Incremental cost-effectiveness analyses result (full incremental analysis)**

|  | **Incremental QALY** | **Incremental Cost (£)** | **ICER** |
| --- | --- | --- | --- |
| Control | 0 | 0 | - |
| Text messages alone | 0.022 | 29 | 1,291 |
| Text messages with financial incentives | 0.031 | 130 | 11,691 |

Note: Some numbers are rounded

**Supplementary Figure 8a: Cost-effectiveness plane**

**Supplementary Figure 8b: Cost-effectiveness acceptability curves**

**Supplementary Figure 9a: Tornado diagram: ICER (Text messages with financial incentives vs Control)**

**Supplementary Figure 9b: Tornado diagram: ICER (Text messages alone vs Control**

**References**

1. Jones KC, Weatherly H, Birch S, et al. Unit costs of health and social care 2022 manual. . 2023.

2. Curtis LA, Burns A. *Unit Costs of Health and Social Care 2015.* Personal Social Services Research Unit; 2015.

3. NHS England. National Cost Collection for the NHS 2021/22. Available at: <https://www.england.nhs.uk/publication/2021-22-national-cost-collection-data-publication/.> Accessed Nov 6, 2023.

4. Public Health Scotland 2019/2020. Specialty costs and activity - inpatients in all specialties (exc long stay), by hospital. Available at: <https://www.isdscotland.org/health-topics/finance/costs/Detailed-Tables/Speciality-Costs/Acute-Medical.asp.> Accessed Nov 6, 2023.

5. Max Planck Institute for Demographic Research, (Germany) U of CB (USA), and FI for DS (France). &nbsp. Human Mortality Database. Available at: [www.mortality.org.](file:///C:\GOS_revision\Resumbmission\www.mortality.org) Accessed Nov 9, 2023.

6. Kent S, Aveyard P, Astbury N, Mihaylova B, Jebb SA. Is doctor referral to a low‐energy total diet replacement program cost‐effective for the routine treatment of obesity? *Obesity*. 2019;27:391–398.

7. Institute for Health Metrics and Evaluation. Health Data Exchange: GBD Results Tool. Available at: <http://ghdx.healthdata.org/gbd-results-tool>.

8. Sullivan PW, Slejko JF, Sculpher MJ, Ghushchyan V. Catalogue of EQ-5D scores for the United Kingdom. *Medical Decision Making*. 2011;31:800–804.

9. Hartmann‐Boyce J, Cobiac LJ, Theodoulou A, et al. Weight regain after behavioural weight management programmes and its impact on quality of life and cost effectiveness: evidence synthesis and health economic analyses. *Diabetes, Obesity and Metabolism*. 2023;25:526–535.

10. Blakely T, Cobiac LJ, Cleghorn CL, et al. Health, health inequality, and cost impacts of annual increases in tobacco tax: multistate life table modeling in New Zealand. *PLoS medicine*. 2015;12:e1001856.

11. Briggs A, Sculpher M, Claxton K. *Decision Modelling for Health Economic Evaluation.* Oup Oxford; 2006.
